# Preparation of Cellulose Nanofiber Aerogels from Paper Sludge Waste and Their Use for the Removal of Metal Ions Used in Batteries from Water

**DOI:** 10.3390/gels12100935

**Published:** 2026-10-17

**Authors:** Wafaa Abou-Elseoud, Shaimaa Fadel, Enas Hassan, Damien Quemener, Karim Aissou, Suming Li, Mohammad Hassan

**Affiliations:** 1 Cellulose and Paper Department, National Research Centre, 33 El-Behouth Street, Dokki, Giza 12622, Egypt; ws.seleim@nrc.sci.eg (W.A.-E.); sm.fadel@nrc.sci.eg (S.F.); ea.elgarhy@nrc.sci.eg (E.H.); 2 Institut Européen des Membranes, IEM, UMR-5635, University of Montpellier, ENSCM, CNRS, 34095 Montpellier, France; damien.quemener@umontpellier.fr (D.Q.); karim.aissou@umontpellier.fr (K.A.); 3 Faculty of Applied Arts, Egyptian Russian University, Badr City 11829, Egypt

**Keywords:** aerogels, nanocellulose, heavy metal adsorption, paper sludge, fiber dust, TEMPO-oxidation, battery industry effluents, kaolin, Purolite

## Abstract

Developing cost-effective, bio-based adsorbents for hazardous battery industry effluents is a major environmental challenge. This study isolated cellulose nanofibers (CNFs) from primary paper sludge (PS)—a tissue manufacturing waste—via chemical modification using (2,2,6,6-Tetramethylpiperidin-1-yl)oxyl oxidation (TEMPO oxidation) and ultrasonication to fabricate highly porous aerogels. The PS-CNF aerogels were evaluated for removing battery-related metal ions (Pb^2+^, Cd^2+^, Co^2+^, Mn^2+^, Ni^2+^, and Li^+^). To assess the structural impact of residual kaolin in the sludge, performance was compared against aerogels derived from fiber dust (FD) waste, as well as a commercial resin (Purolite C-100). The PS-CNF aerogels demonstrated superior maximum adsorption capacities of 39.28 mg/g for Pb^2+^, 29.80 mg/g for Cd^2+^, 21.05 mg/g for Co^2+^, 20.19 mg/g for Mn^2+^, 16.80 mg/g for Ni^2+^, and 11.33 mg/g for Li^+^. They consistently outperformed the FD-CNF matrix across the entire series. This enhanced efficiency is attributed to the residual kaolin in the paper sludge, which also acts as a physical spacer, increasing the aerogel’s internal porosity. Adsorption generally followed the Langmuir isotherm and pseudo-second-order kinetics. Furthermore, the PS-CNF aerogels exhibited higher efficiency than the commercial Purolite C-100 cation exchange resin. Ultimately, this work demonstrates the potential of upcycling paper sludge into sustainable, high-performance adsorbents for treating toxic battery effluents.

## 1. Introduction

Rapid global industrialization and the concomitant generation of wastewater mean that the development of cost-effective, high-efficiency water purification technologies is crucial if we are to preserve scarce freshwater resources. Industrial effluents are highly diverse and chemically complex, necessitating tailored materials and methodologies for selective pollutant removal. Specific attention is paid to the removal and recovery of hazardous and economically valuable metal ions (e.g., cobalt, nickel, zinc, manganese, lead, and cadmium) widely used in commercial batteries (e.g., lithium-ion, zinc–carbon, nickel–cadmium) from aqueous effluents, as these species pose well-documented environmental and human health risks [1,2]. Exposure to these metal ions, either in high doses or in low doses for prolonged periods, induces severe cellular damage predominantly through oxidative stress and the displacement of essential biological elements. Neurologically, Pb, Mn, and Li provoke profound toxicity, ranging from cognitive deficits and fatal encephalopathy to severe motor dysfunctions such as Parkinson-like “manganism” [3,4,5,6]. Systemically, Cd, Pb, and Li aggressively target the renal system, causing progressive chronic kidney failure [7,8,9], with Cd uniquely driving extreme bone demineralization [10]. Furthermore, systemic accumulation of Co and Ni triggers severe cardiotoxicity, multi-organ endocrine disruption, and respiratory diseases [11,12], while both Ni and Cd are classified as potent Group 1 human carcinogens [13,14]. Additionally, Pb heavily impairs heme biosynthesis, leading to severe systemic anemia [15].

Various conventional techniques are employed for metal remediation [16], including membrane processes (e.g., electrodialysis [17], reverse osmosis [18], ultrafiltration, and nanofiltration [19,20]), solvent extraction [21], electrochemical treatment [22], chemical precipitation [23], adsorption technology [24]. Among these, adsorption technology stands out due to its operational simplicity, scalability, high removal efficiency, and favorable techno-economic and environmental profile (e.g., low energy demand, absence of chemical precipitants, and closed-loop potential) [16,25]. Because the adsorbent is the core component governing system performance and viability, cost and adsorption capacity are critical selection criteria. In this context, lignocellulosic-based adsorbents represent a particularly attractive class of materials owing to their sustainability, low cost, and biocompatibility [26,27]. Furthermore, nanostructuring these bio-based sorbents has emerged as a promising strategy, as the introduction of nanoscale features dramatically enhances specific surface area and active site accessibility, thereby significantly boosting adsorption capacity [28,29].

Owing to these advantages, CNFs isolated from various lignocellulosic sources have been extensively investigated in recent years for water purification, particularly for the removal of heavy metal ions, such as Pb (II), As (III), Cr (VI), Cr (III), Cu (II), and Cd (II) following surface functionalization [30]. CNFs can be fabricated into gels, films, and aerogels for water treatment applications. The aerogel form of CNFs is characterized by a highly porous 3D architecture, low density, good mechanical properties, and open pore network for high mass transfer/diffusion of contaminants [31]. To date, however, the primary bottleneck remains its high production cost, which encompasses the energy- and chemical-intensive pulping and bleaching stages required to produce purified pulp, followed by the mechanical or chemo-mechanical isolation of the nanofibers themselves [32].

A viable route toward reducing the production cost of CNFs involves valorizing fiber-rich paper industry waste streams. Among these, primary paper sludge—generated during the screening, clarification, and dissolved air flotation (DAF) stages of paper recycling—is of particular interest. This residue contains substantial amounts of cellulosic fines alongside inorganic fillers. Historically, paper sludge has represented a negative-value waste, as mills incur significant disposal fees for landfilling. Furthermore, the exact composition and properties of primary sludge vary widely depending on the mill operations, particularly whether it originates from virgin pulping/bleaching lines or recycled de-inking facilities [33] Primary paper sludge derived from paper recycling and de-inking operations poses the greatest utilization challenge due to its high ash content—stemming from inorganic fillers—alongside residual inks and carbonaceous matter deeply embedded within the fibrous network. However, suitable de-ashing treatments effectively concentrate the cellulose fraction, enabling its valorization for higher-value applications such as preparation of calcium formate [34], hydrolysis into simple sugars [35,36], and nanocellulose production [37]. Because paper sludge already consists of processed cellulose fibers, it eliminates the capital and chemical expenses associated with virgin pulping and bleaching—steps otherwise mandatory when using wood or agricultural residues. Furthermore, these fibers have already undergone mechanical refining during original paper manufacture, significantly reducing the energy input required for subsequent nanofibrillation [15]. Driven by these advantages, relatively few studies have investigated the isolation of CNF from paper sludge across diverse industrial sources using various processing routes. For instance, Parit et al. used kraft sludge fibers—including unbleached fractions—for isolation of CNF via TEMPO-mediated oxidation [38]. Sardir et al. followed enzymatically assisted centrifugation approach for isolation of CNF [39], and Mautner et al. used ultrafine grinding for the same purpose [40]. Li et al. used highly refined fiber fractions derived from tissue and towel manufacturing for isolation of CNF by enzymatic treatment [41], while Gallo Stampino et al. used chemical (TEMPO-mediated oxidation) [42], and Adu et al. used direct ultrafine grinding [43,44] for isolation of CNF from similar fiber sources. Meanwhile, secondary fibers from mixed and recycled paper sludges—spanning diverse ash contents— have been utilized by Du et al. and Altay et al. for isolating CNF through chemical or enzymatic pretreatment followed by high-shear mechanical disintegration, such as microfluidization [45,46].

Despite the diverse applications explored for sludge-derived CNF, its utility in heavy metal remediation remains virtually unaddressed. To the best of our knowledge, CNF isolated from sludge has only been investigated by Mautner et al. for the adsorption of Cu(II) ions [40]. In that work, the sludge used was from dissolving cellulose pulp residues, which is a highly pure cellulose (95% cellulose); after CNF isolation by ultrafine grinding, phosphorylation allowed the nanofibers to be used for adsorption of Cu.

Therefore, this study aims to evaluate aerogels fabricated from CNF for the removal of battery-relevant heavy metal ions from aqueous solutions. The CNF was isolated from primary paper sludge generated during the recycling of writing and printing paper, following acid de-ashing (calcium carbonate removal) and subsequent TEMPO-mediated oxidation coupled with ultrasonication. To elucidate the effect of residual kaolin in paper sludge on adsorption performance, the results were compared to aerogels derived from fiber dust—a waste stream from the tissue paper industry consisting of pure paper pulp—processed under identical isolation conditions. Furthermore, both CNF aerogel samples were thoroughly characterized to assess structural differences stemming from the two distinct feedstocks.

## 2. Results and Discussion

### 2.1. Paper Sludge and Fiber Dust Used

In the current work, two waste materials from tissue paper manufacturing were used to isolate CNFs: paper sludge and fiber dust. The sludge used was the primary paper sludge produced from the de-inking and recycling of writing and printing paper. Due its high ash content (~37%), especially calcium carbonate filler, it was treated with dilute formic acid to remove the calcium carbonate [34]. After this de-ashing step, a considerable amount of the kaolin remained within the fibers because it resisted the acid treatment. The second waste used (fiber dust) is generated during the trimming of tissue paper reels and captured by air filters. It is essentially a bleached paper pulp in dust form. The chemical analysis revealed that the fiber dust consisted predominantly of α-cellulose (70.49%) and hemicelluloses (25.40%) with minimal ash (1.48%), lignin (1.67%), and extractives (0.80%), whereas the formic acid-treated paper sludge contained lower amounts of α-cellulose (54.76%) and hemicelluloses (21.17%) alongside significantly higher levels of acid non-hydrolyzable organics (9.80%), extractives (2.95%), and ash (11.87%) consisting mainly of kaolin [34].

### 2.2. Isolation and Characterization of TEMPO-Oxidized CNF from Paper Sludge and Fiber Dust

**Figure 1:**
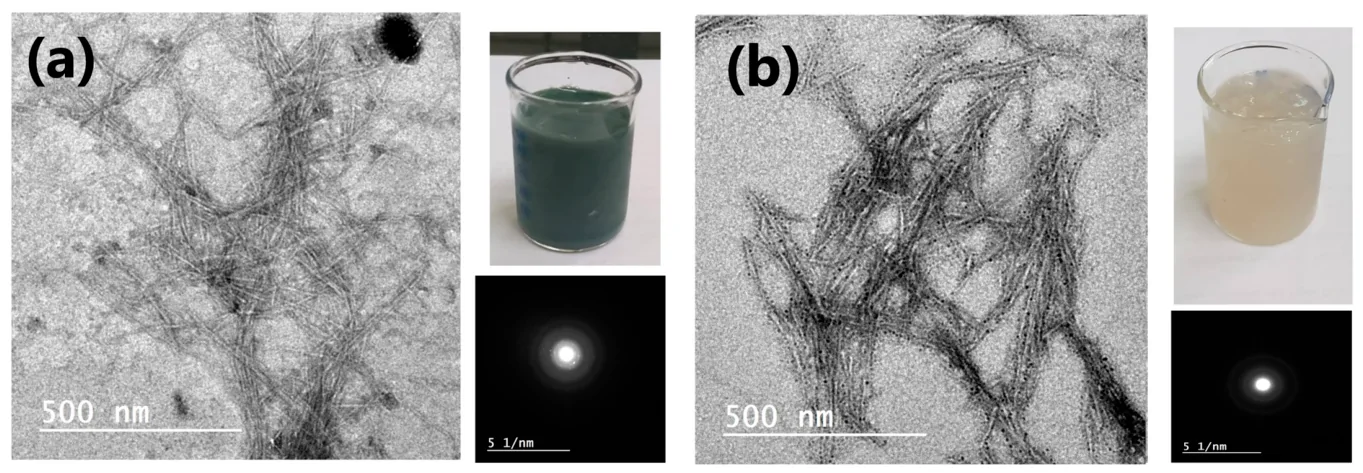
TEM images of TEMPO-oxidized CNF isolated from (**a**) de-ashed paper sludge fibers and (**b**) fiber dust.

To isolate CNF from the de-ashed paper sludge and fiber dust, TEMPO-mediated oxidation was employed. This well-established treatment introduces carboxylic groups at the C-6 primary hydroxyls that swell the cellulose fibers, facilitating their mechanical breakdown [47,48]. Following the oxidation process, the fiber dust achieved a higher carboxylic group concentration (0.55 mmol/g) than the paper sludge (0.475 mmol/g). This difference directly reflects the higher initial α-cellulose proportion present in the fiber dust. Because of the combined effects of the TEMPO-induced swelling and the inherently short length of these waste fibers, short ultrasonic treatment was required to rapidly disintegrate the oxidized materials into CNF. Transmission electron microscopy (TEM) confirmed the successful isolation of elementary cellulose fibrils for both waste streams, revealing nanofibers approximately 4 to 6 nm in width (Figure 1). Furthermore, TEM imaging of the sludge-derived CNF detected nanoscale aggregates of the residual kaolin ash; the small size of these impurities suggests the ash particles were simultaneously broken down during the ultrasonic treatment. The Selected Area Electron Diffraction (SAED) pattern shown in image 1a displays sharp, bright diffraction spots corresponding to highly crystalline kaolin superimposed over the broad, diffuse halos of the semi-crystalline cellulose nanofiber matrix. The zeta potential measurement of the CNF suspensions showed a mean ζ-potential of −45 and −49.5 mV for the FDA-CNF and PS-CNF suspension, respectively. The lower ζ-potential of the PS-CNF in spite of its lower carboxylic content could be attributed to the presence of residual kaolin, which is alkali-activated by the alkaline medium (pH 10) during TEMPO-oxidation.

**Figure 2:**
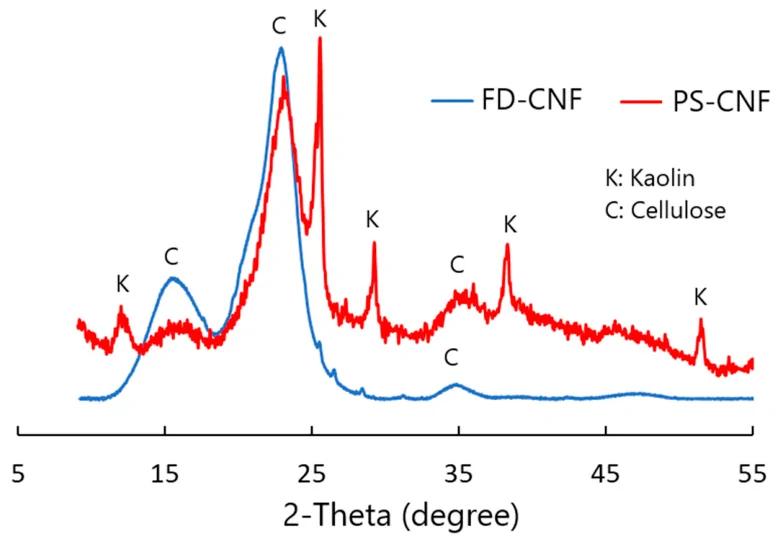
XRD patterns of PS-CNF and FD-CNF.

The presence of residual kaolin within the CNF was also proven by the X-ray diffraction (XRD) patten of paper sludge CNF (PS-CNF) compared with that of FD-CNF (Figure 2). The peaks at 2θ = 16, 23, and 35° are due to diffraction from the (110), (200), and (004) planes of cellulose, respectively [49] while the peaks at 2θ = 25.5°, 29.3°, 38.4°, and 51.5° are attributed to the residual kaolin [50,51], which is usually added as a filler to writing and printing paper products. It should be pointed out that the residual kaolin in the oxidized CNF was subjected to both acidic pH during the de-ashing using formic acid and to an alkaline medium during TEMPO-mediated oxidation. The XRD pattern of kaolin without these pretreatments has a kaolinite structure with peaks at 12.24° (main peak), 24.6°, 26°, 28.3°, 37.4°, 38.2°, 39.0°, 45.2°, and 50.7° due to the aluminum silicate structure of Al_2_Si_2_O_5_(OH)_4_ [50]. After the de-ashing and TEMPO-mediated oxidation, only peaks at 2θ = 25.5°, 29.3°, 38.4°, and 51.5° were detected in the XRD pattern.

### 2.3. Preparation and Characterization of CNF Aerogels

**Figure 3:**
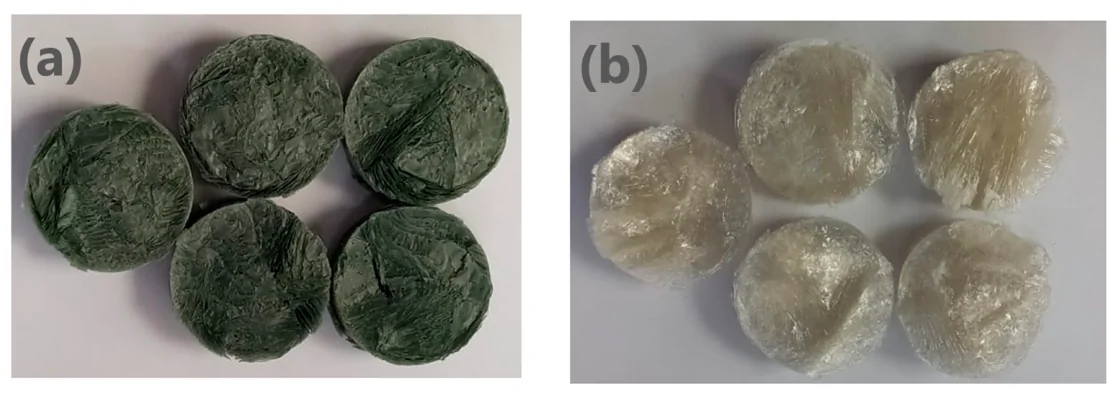
Aerogels made from (**a**) PS-CNF and (**b**) FD-CNF.

Aerogels from TEMPO-oxidized CNF derived from paper sludge (PS-CNF) and fiber dust (FD-CNF) were successfully fabricated via freeze-drying (Figure 3). The bulk densities of the PS-CNF and FD-CNF aerogels were 0.0490 ± 0.0016 g/cm^3^ and 0.0338 ± 0.0004 g/cm^3^, with corresponding porosities of 96.71 ± 0.11% and 97.41 ± 0.02%, respectively. The higher bulk density observed for PS-CNF is primarily attributed to the high skeletal density of the residual kaolin (~12%). Nevertheless, both aerogels maintained remarkably high and comparable porosities, demonstrating that the presence of mineral filler did not compromise the formation of the open cellular macroporous network. The aerogels were further evaluated in terms of their specific surface area, microscopic structure, and compressive mechanical properties.

#### 2.3.1. Brunauer–Emmett–Teller (BET) Surface Area and Porosity Characteristics

The surface area and porosity characteristics of the prepared aerogels are shown in Table 1; the BET curves, total pore volume and average pore size data are shown in the Supplementary Figures S1 and S2. As the results show, both types of the aerogels had similar surface area, total pore volume, and average pore size despite the presence of kaolin residues (~12%) in the case of the PS-CNF aerogel. The BET analysis confirms that the walls of the aerogel scaffolds possess a dense internal microporous network, which provides high specific surface area (~270 m^2^/g). The surface area of both the PS-CNF and FD-CNF aerogels is high; the reported surface area of aerogels prepared from CNF in previous studies ranged from 60 to 350 m^2^/g [52].

#### 2.3.2. Scanning Electron Microscopy (SEM) Investigation

**Figure 4:**
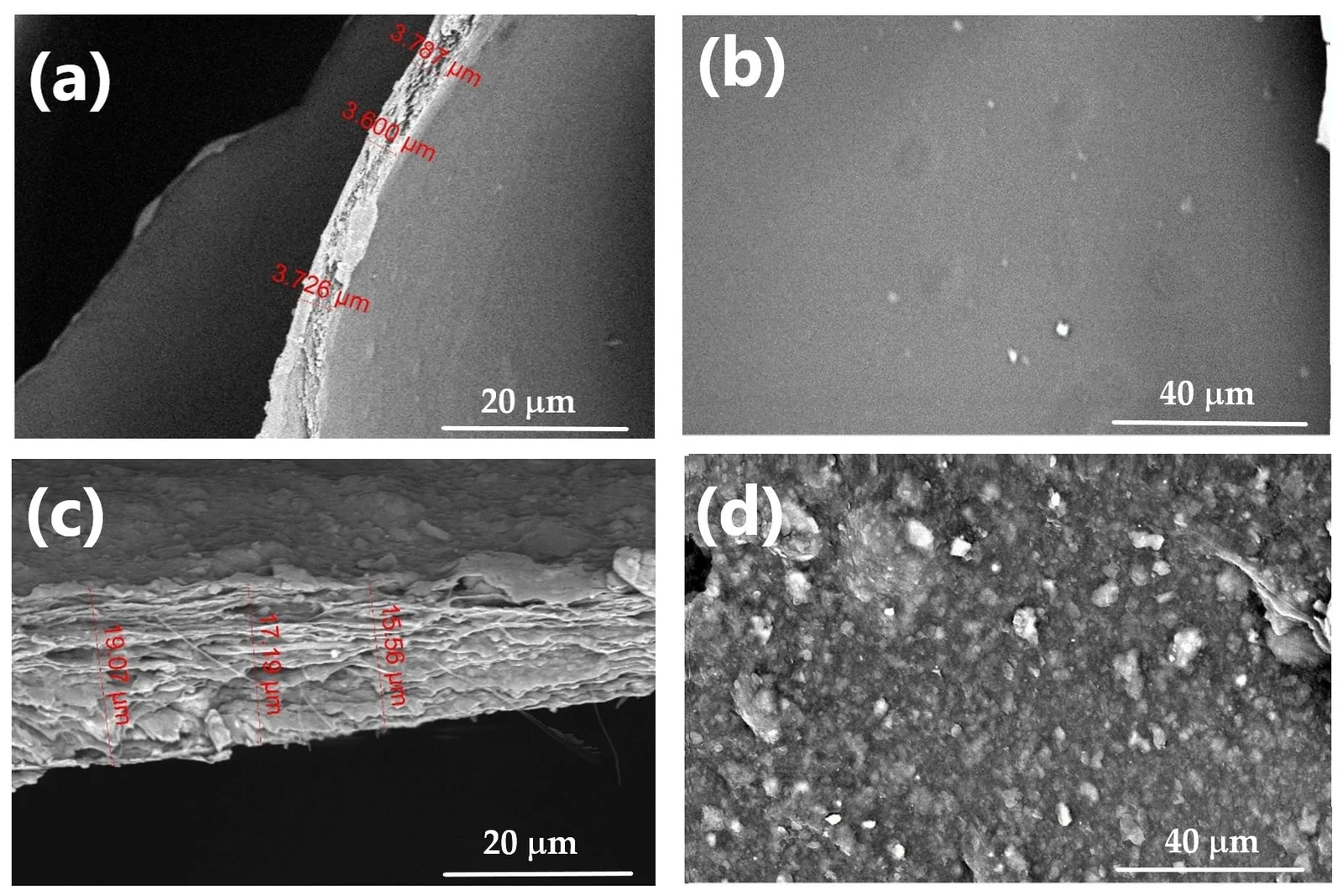
Cross-section and surface SEM images of aerogels walls made from FD-CNF (**a**,**b**) and PS-CNF (**c**,**d**).

The SEM images of the CNF aerogels showed a regular structure with relatively large voids (Figure 4). Furthermore, images of the aerogel walls at higher magnification show that the thicknesses of the PS-CNF and FD-CNF laminar walls clearly differ. The thickness of FD-CNF was ~4 μm, while that of PS-CNF was ~9–17 μm. This increase indicates that the presence of residual kaolin within the nanocellulose fibrils promoted an expanded multi-layered structure; residual kaolin particles acted as inorganic spacers/fillers during assembly and freeze-drying, and by intercalating between the cellulose nanofibrils (CNFs), kaolin prevented tight self-aggregation and hydrogen bonding (hornification) of the CNF sheets. Furthermore, the cross-sectional surface of PS-CNF appeared rough with embedded kaolin particles, while FD-CNF exhibited a much smoother morphology. Despite this pronounced difference in apparent wall thickness, BET analysis yielded nearly identical specific surface areas for FD-CNF (269.4 m^2^/g) and PS-CNF (271.1 m^2^/g) as shown above. It should be noted that in the PS-CNF aerogel, the presence of residual kaolin filler (~12%)—which possesses a much higher density and lower porosity than the cellulose aerogel—affected the calculated BET surface area, explaining the similar BET values despite the clear differences observed in the SEM images.

**Figure 5:**
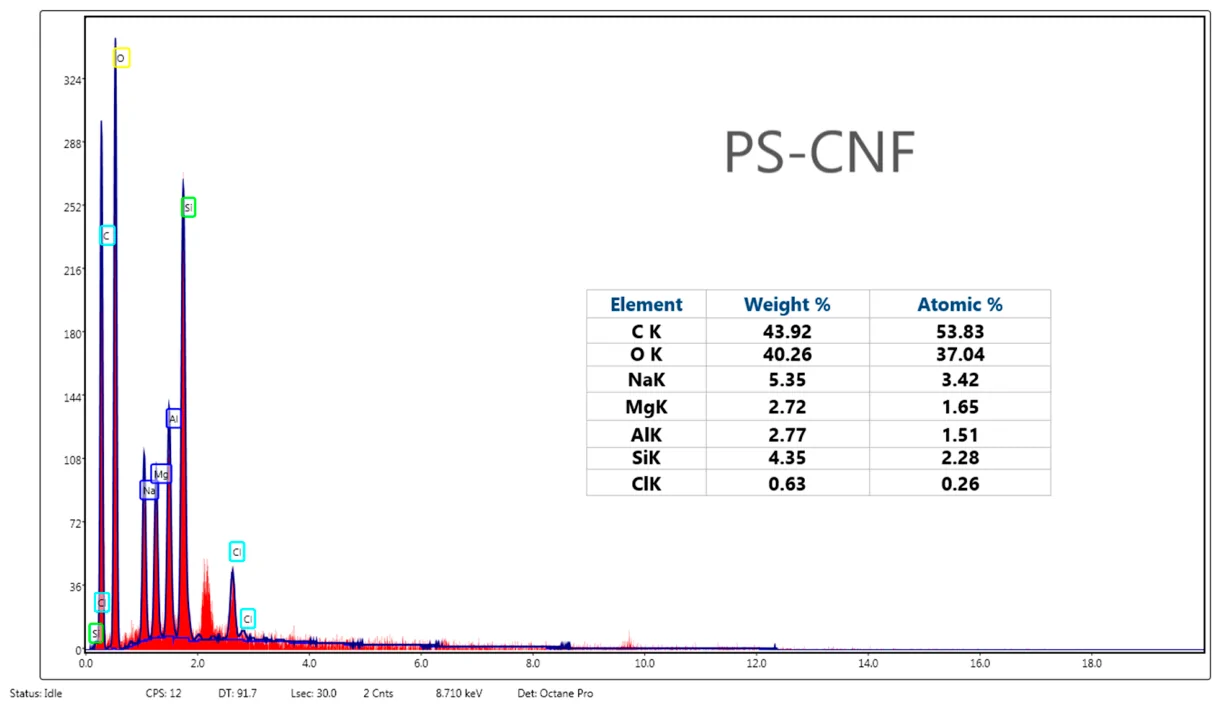

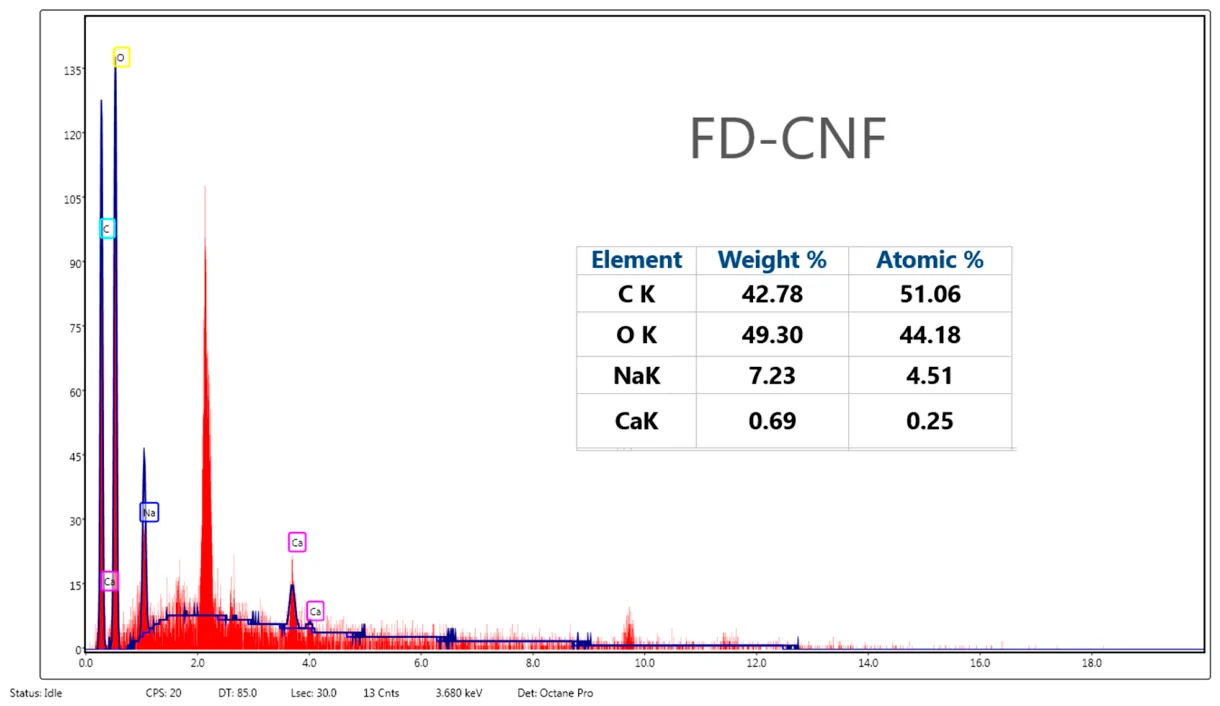
EDX analysis of PS-CNF and FD-CNF aerogels.

The energy-dispersive X-ray spectroscopy (EDX) analysis of the PS-CNF aerogel showed the presence of residual kaolin, as evidenced by the presence of signals for Si, Al, and O (Figure 5). In addition, the analysis showed the presence of Mg, which could be due to the presence of talc powder also being used as a filler. Since talc is a magnesium silicate-based material, while kaolin is an aluminum silicate-based material, the ratio detected in the EDX is not perfectly matched to the theoretical Al:Si ratio of kaolin. The presence of Na could be attributed to the exchange of hydrogen protons with the hydroxy groups of kaolin, as well as the hydroxy and carboxylic groups of oxidized cellulose with Na ions as a result of the alkaline pH during TEMPO-mediated oxidation. The EDX spectrum also showed major signals of C and O of the cellulosic fibers (Figure 5). It is important to mention that kaolin in the paper sludge is much more abundant than talc, as shown by the XRF analysis of the residual ash in the de-ashed paper sludge, which is presented in Table 2 [34].

#### 2.3.3. Mechanical Properties of FD-CNF and PS-CNF Aerogels

**Figure 6:**
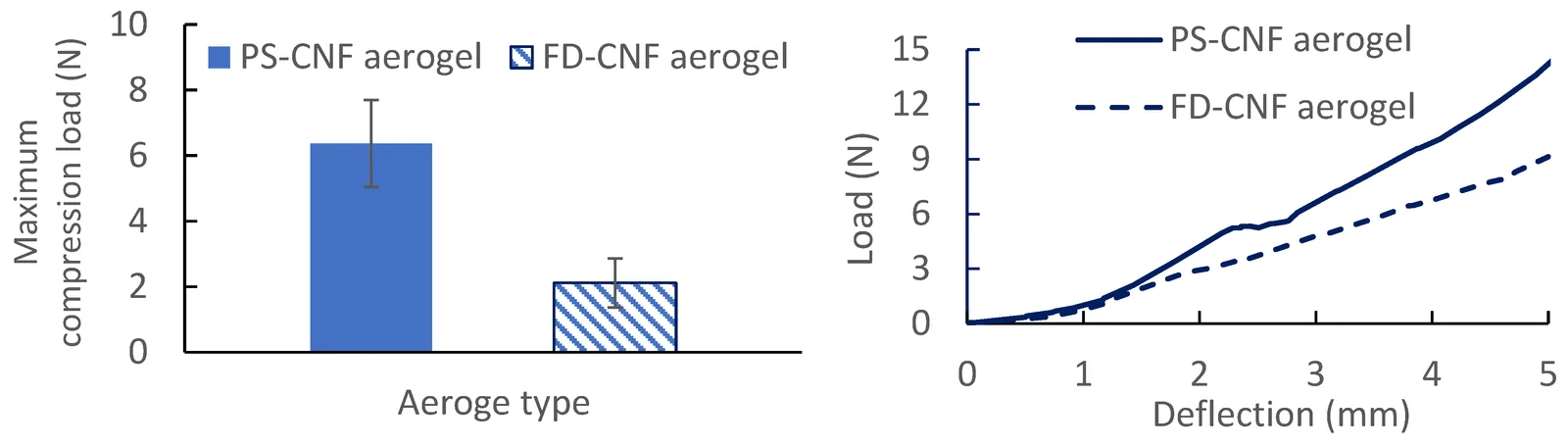
Compression strength testing results and load/deflection curves of PS-CNF and FD-CNF aerogels during the test.

The compression strength of the prepared aerogels was tested, and the results are shown in Figure 6. As the figure shows, the response of the aerogels to the compression force and the maximum force withstood by them before deformation was higher in the PS-CNF aerogel than FD-CNF. The maximum compression strength before compacting the aerogel was 6.37 ± 1.23 and 2.11 ± 0.75 for PS-CNF aerogel and FD-CNF, respectively. The higher stiffness and compression strength of the PS-CNF aerogel than the FD-CNF aerogel could be due the presence of residual kaolin filler in the PS-CNF aerogel.

### 2.4. Metal Ion Adsorption by PS-CNF and FD-CNF Aerogels

The adsorption capacity of the prepared PS-CNF and FD-CNF aerogels for several metal ions used in the battery industry (Pb, Cd, Co, Ni, Mn, and Li) was evaluated. The effects of metal ion concentration, contact time, and temperature on the adsorption are discussed in the following sections. All experiments were conducted at the natural pH of the as-prepared solutions (Table 3).

The pH of the aqueous solution is a critical parameter governing the adsorption process. At highly acidic pH values, the adsorption capacity is significantly restricted due to the high concentration of H^+^ ions, which compete with the heavy metal cations for available active binding sites on the aerogel surface. On the other hand, at highly alkaline pH values, the metal ions tend to undergo hydrolysis and precipitate as insoluble metal hydroxides, restricting true surface adsorption [53].

#### 2.4.1. Effect of Metal Ions Concentration on Adsorption

**Figure 7:**
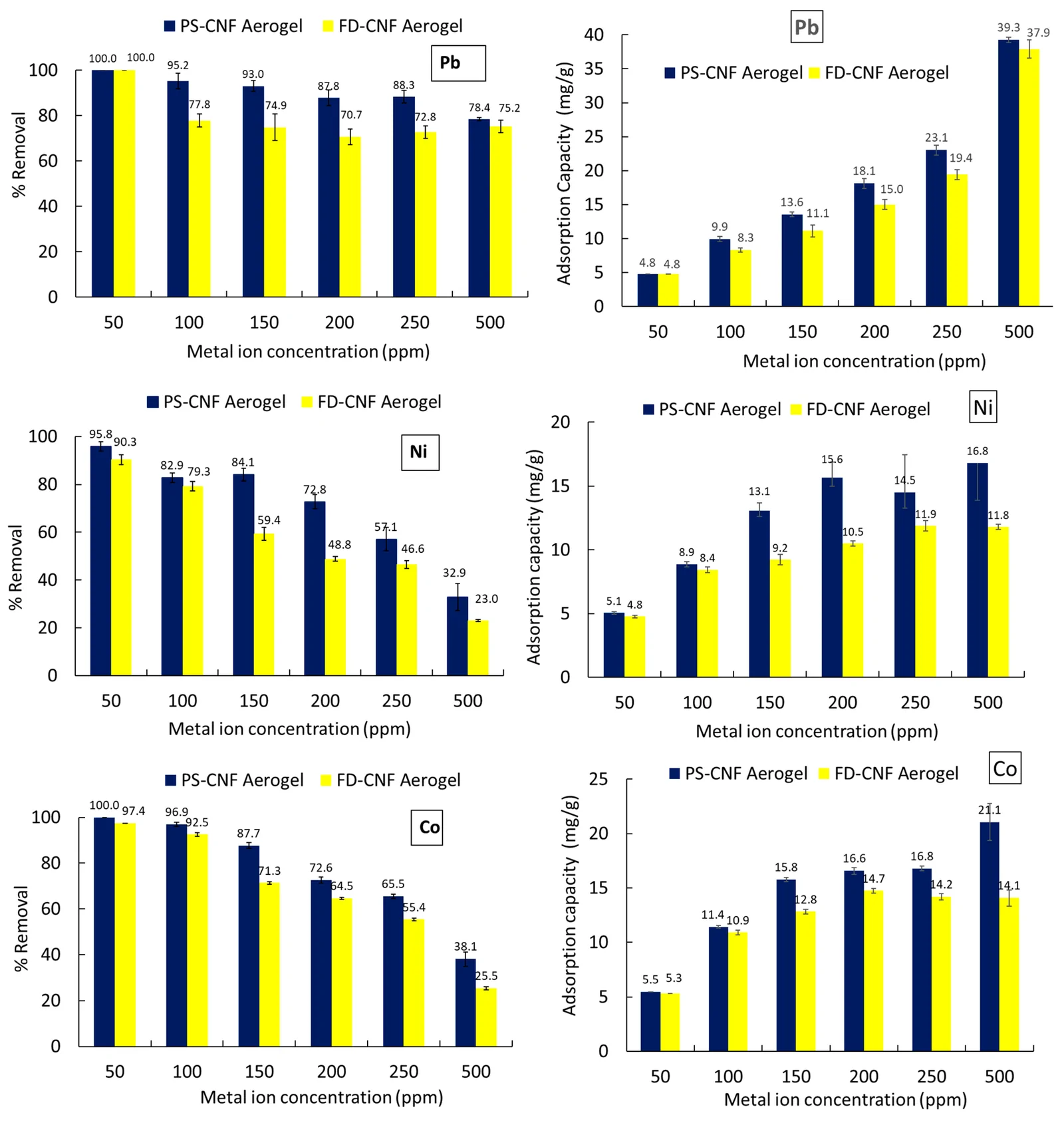

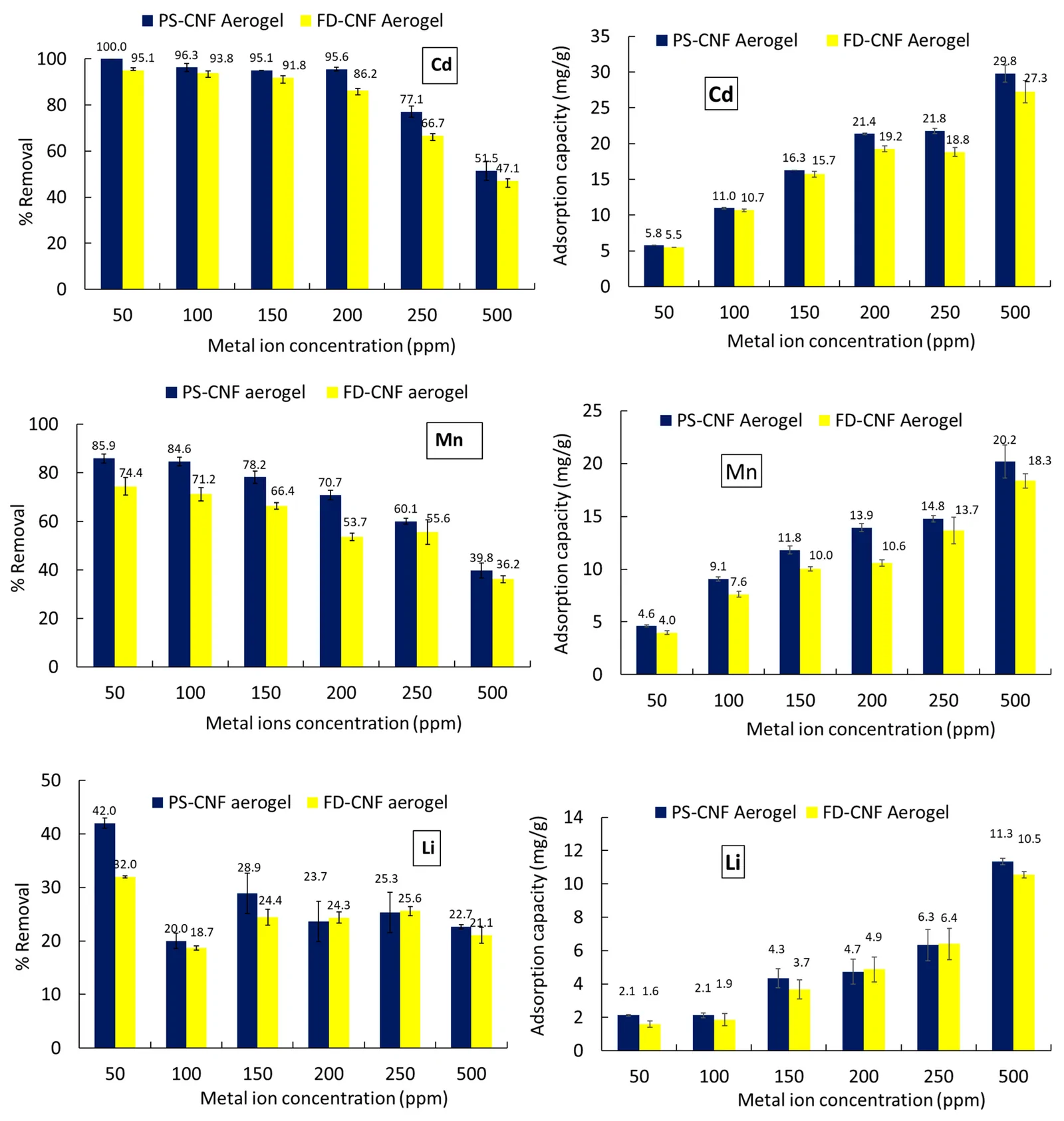
Effect of concentration of Pb, Ni, Co, Mn, Cd, and Li metal ions on the adsorption by PS-CNF and FD-CNF aerogels.

Adsorption experiments were conducted using initial metal ion concentrations ranging from 50 to 500 ppm, with a fixed contact time of 1 h. The adsorption performance of aerogels prepared from PS-CNF was compared to those derived from fiber dust, or FD-CNF. Figure 7 illustrates the effect of initial metal ion concentration on the adsorption capacity of the CNF aerogels. The adsorption performance of PS-CNF and FD-CNF aerogels demonstrates distinct concentration-dependent behaviors and metal-specific affinities across the studied metal ions.

The effect of initial metal ion concentration on the adsorption performance of PS-CNF and FD-CNF aerogels demonstrates a consistent thermodynamic mechanism across all tested heavy metals (Pb, Ni, Co, Cd, Mn) and lithium (Li). For all evaluated metal ions, the percentage removal reaches its maximum at the lowest initial concentration of 50 ppm. At this baseline, PS-CNF achieves near-complete or highly efficient removal for several metals, approaching 100% for Pb, Cd, and Co and exceeding 95% for Ni, while Li showed the lowest adsorption (~42%). As the initial concentration increases toward 500 ppm, the removal efficiency steadily declines for all metals. This inverse relationship occurs because at low initial concentrations, there is an abundance of accessible active binding sites on the aerogel surface relative to the solute molecules; as the metal ion concentration increases, these functional sites rapidly saturate, leaving a progressively larger fraction of non-adsorbed ions in water [54]. Conversely, the equilibrium adsorption capacity (mg/g) increases progressively as the initial concentration rises from 50 ppm to 500 ppm for both aerogel types. A higher initial metal ion concentration establishes a stronger concentration gradient between the bulk solution and the solid adsorbent phase. This elevated gradient acts as a thermodynamic driving force that overcomes mass transfer resistance, pushing more metal ions into the porous network of the aerogels and maximizing the utilization of internal binding sites [55].

When comparing the two adsorbents, the PS-CNF aerogel consistently exhibits better adsorption values than the FD-CNF aerogel across the majority of concentration intervals and metal types. At the maximum tested concentration of 500 ppm, PS-CNF achieves slightly higher total adsorption capacities than FD-CNF for Pb (~39 mg/g vs. ~38 mg/g), Ni (~17 mg/g vs. ~12 mg/g), Co (~21 mg/g vs. ~14 mg/g), Cd (~30 mg/g vs. ~27 mg/g), Mn (~20 mg/g vs. ~18 mg/g), and Li (~11 mg/g vs. ~10.5 mg/g). This consistent outperformance indicates that the cellulose nanofiber network derived from paper sludge possesses a more optimized porous morphology or a higher density of accessible, reactive functional groups capable of coordinating with cationic contaminants.

The concentration-dependent data also establishes a distinct hierarchy in the binding affinity of the aerogels toward different specific metal ions. Based on the peak adsorption capacities achieved at 500 ppm by the PS-CNF aerogel, the affinity sequence is Pb (~39 mg/g) > Cd (~30 mg/g) > Co (~21 mg/g) ≈ Mn (~20 mg/g) > Ni (~17 mg/g) > Li (~11 mg/g). This preferential binding to heavy metals over lighter alkaline metals is dictated by the intrinsic chemical properties of the cations. Oxygen-rich functional groups on the TEMPO-oxidized cellulose matrix (such as hydroxy and carboxylic groups) act as electron donors and form stronger coordinate chelating bonds with target cations that possess higher electronegativities and larger ionic radii [56]. Consequently, heavy metals like Pb (electronegativity = 2.33) form highly stable, specific complexes with the aerogel surface, whereas lighter alkali metals like Li (electronegativity = 0.98) participate primarily through weaker electrostatic interactions and ion exchange, resulting in noticeably lower equilibrium adsorption capacities [57].

The enhanced adsorption capacity of the PS-CNF aerogel relative to the FD-CNF variant is partially attributable to its distinct microscopic structure. As evidenced by SEM analysis, the PS-CNF aerogel exhibits thicker laminar walls, which suggests that less fibril aggregation occurs during freeze-drying.

Additionally, the residual kaolin within the PS-CNF structure likely contributes to metal ion adsorption. Prior studies have demonstrated that both natural and modified kaolin effectively remove various heavy metal ions, such as Pb, Cu, Mn, and Cd [58,59,60].

Table 4 summarizes the maximum experimental adsorption capacities of the PS-CNF and FD-CNF aerogels for the various metal ions investigated. The adsorption capacity of TEMPO-oxidized CNF depends primarily on the surface-generated carboxylic groups. In the current work, the carboxylic acid content of PS-CNF and FD-CNF was 0.475 and 0.55 mmol/g, respectively. Given the 2:1 stoichiometric binding ratio of carboxylic groups to divalent metal ions (i.e., two -COOH to one M^2+^), the theoretical maximum divalent metal uptake—assuming all -COOH groups remain accessible after freeze-drying—is 0.238 mmol/g for PS-CNF and 0.275 mmol/g for FD-CNF [61]. On the other hand, Li^+^ ions can exchange with the -OH and -COOH groups at a 1:1 stoichiometric ratio in the TEMPO-CNF [62].

As shown in Table 4, for the FD-CNF aerogels, the maximum adsorption of divalent ions (in mmol/g) generally aligned with theoretical expectations, with the exception of Mn. However, for the PS-CNF aerogels, the experimental adsorption of Ni, Co, Cd, and Mn ions exceeded the available carboxylic binding sites. This discrepancy strongly supports the active participation of residual kaolin in adsorbing the studied metal ions. It is important to note that the residual kaolin underwent an acidic treatment during paper sludge de-ashing with formic acid, followed by an alkaline pH during TEMPO-mediated oxidation. Both the acidic treatment [63,64] and alkali treatment with NaOH [60] can improve the capacity of kaolin to adsorb different metal ions such as Cd and Pb ions.

In the case of Pb adsorption, the maximum capacity fell below the expected theoretical value. This reduction is likely due to the significantly larger ionic radius of Pb^2+^ compared to the other divalent ions, leading to increased steric hindrance during adsorption onto the CNF network or kaolin particles.

The adsorption capacities of the prepared PS-CNF and FD-CNF aerogels for Pb, Cd, and Ni ions are higher than those of previously reported TEMPO-CNFs prepared from wood pulp, which exhibited maximum adsorption capacities of 9.7 mg/g, 9.4, mg/g, and 8.6 mg/g for Cd, Pb, and Ni, respectively [52]. A similar capacity for adsorption of Co ions by TEMPO-CNF prepared from wood pulp (~22.27 mg/g) has been reported before [65]. On the other hand, a previous study on glycidyl methacrylate-grafted and sulfonated cellulose microspheres (with an 8% sulfur content and a 63% degree of grafting) demonstrated a maximum adsorption capacity of 16 mg/g for Li^+^ ions [66]. In another study, a cellulose nanocrystals/manganese oxide composite demonstrated a maximum Li^+^ adsorption capacity of 6.30 mg/g [67].

Finally, these results indicate good aqueous wetting of both PS-CNF and FD-CNF aerogels, suggesting that no significant hornification occurred upon freeze-drying [52].

##### Isotherm Modeling of Metal Ion Adsorption

The adsorption data on the effect of metal ion concentration was fitted to the Langmuir, Freundlich, and Dubinin–Radushkevich (D-R) models to investigate the binding mechanisms of PS-CNF and FD-CNF aerogels and to determine their maximum adsorption capacity.

The linear form of the Langmuir isotherm is expressed by Equation (1) [68]:

(1) $$\frac{C_{e}}{q_{e}}=\frac{1}{q_{m}\;K_{L}}+\frac{C_{e}}{q_{m}}$$

where *q_e_* is the equilibrium adsorption capacity (mg/g), *C_e_* is the equilibrium concentration of the metal ions in solution (mg/L), *q_m_* is the maximum monolayer adsorption capacity, and *K_L_* is the Langmuir affinity constant related to the free energy of adsorption (L/mg).

The linear form of the Freundlich isotherm is expressed by Equation (2) [68]:

(2) $$\ln(q_{e})=\ln(K_{F})+\frac{1}{n}\ln(C_{e})$$

where *q_e_* is the equilibrium adsorption capacity (mg/g), *C_e_* is the equilibrium concentration of the metal ions in solution (mg/L), *K_F_* is the Freundlich constant, and n is the heterogeneity factor.

The linear form of the Dubinin–Radushkevich (D-R) isotherm equation is expressed by Equation (3) [69]:

(3) *ln*(*q_e_*) = *ln*(*q_m_*) − *K_DR_*ε^2^

*ε* is calculated from Equation (4):

(4) $$\varepsilon =\mathrm{RT}\;\ln(1+\frac{1}{C_{e}})$$

where *q_e_* is the equilibrium adsorption capacity (mg/g), *q_m_* is the theoretical monolayer saturation capacity (mg/g), *K_DR_* is Dubinin–Radushkevich isotherm constant, *ε* is the Polanyi potential, *R* is the universal gas constant, *T* is the absolute temperature (in Kelvin), and *C_e_* is the equilibrium concentration of the adsorbate (mg/L).

The Langmuir model assumes a perfectly homogeneous surface where adsorption occurs as a single monolayer, terminating when all identically energetic active sites are occupied (establishing a maximum theoretical capacity, *q_m_*). In contrast, the Freundlich model describes multilayer adsorption on a highly heterogeneous surface, where binding sites possess varying affinities and there is no assumed saturation limit. Meanwhile, the D-R model is based on the pore-filling theory rather than layer-by-layer surface coverage; it accounts for non-uniform adsorption potential and is distinctly valuable for calculating the mean free energy of adsorption (*E*) in order to determine whether the adsorption process is primarily physical or chemical [68,69].

The results of fitting the experimental data to the Langmuir, Freundlich, and Dubinin–Radushkevich (D-R) models are listed in Table 5. PS-CNF and FD-CNF having higher Langmuir correlation coefficients (*R*^2^) than Freundlich correlations suggests that monolayer adsorption occurs on homogeneous surfaces with equivalent site affinities and negligible adsorbate interactions [69]. The exception to this trend was observed with Pb ions. This deviation is likely attributable to the significantly larger ionic radius and steric hindrance of Pb^2+^ compared with the other metal ions, which restricts access to adjacent active sites and prevents complete uniform saturation. Consequently, the adsorption behavior of Pb is more accurately described by the Freundlich isotherm model (R^2^ = 0.96–0.99), which accounts for heterogeneous binding without assuming strict monolayer saturation. Additionally, the relatively low R^2^ values obtained when modeling Li ion adsorption data could be attributed to the simultaneous interaction of Li^+^ with both the carboxylate and the numerous hydroxyl groups on the cellulose backbone, as well as with the residual kaolin. The involvement of multiple distinct binding sites creates a heterogeneous adsorption environment, making uniform surface saturation difficult to achieve.

The calculated Langmuir separation factors (*R_L_*) for all ions, alongside the Freundlich heterogeneity factors (1/*n*), ranged between 0 and 1, indicating a highly favorable adsorption process.

The relatively lower *R*^2^ values associated with the D-R model across most samples suggest that micropore volume filling was not the primary driving mechanism, implying that the macromolecular structure of the PS-CNF and FD-CNF aerogels was sufficiently open to diminish the restrictive effects of pore size limitations.

#### 2.4.2. Effect of Contact Time on Metal Ion Adsorption

**Figure 8:**
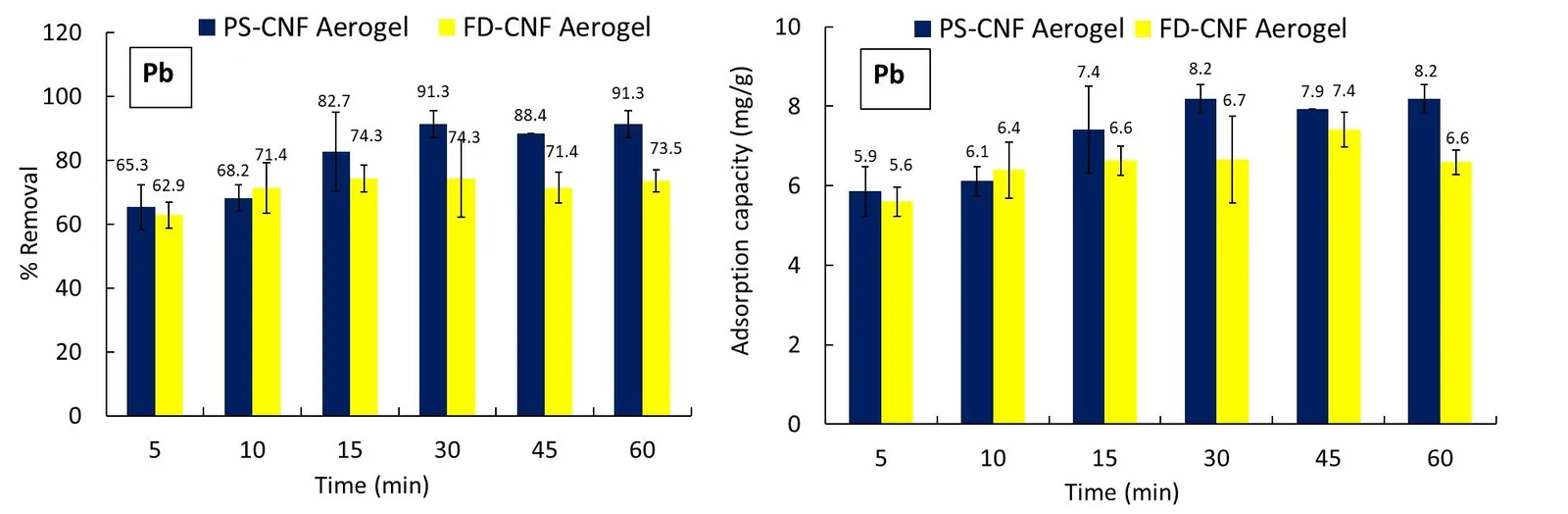

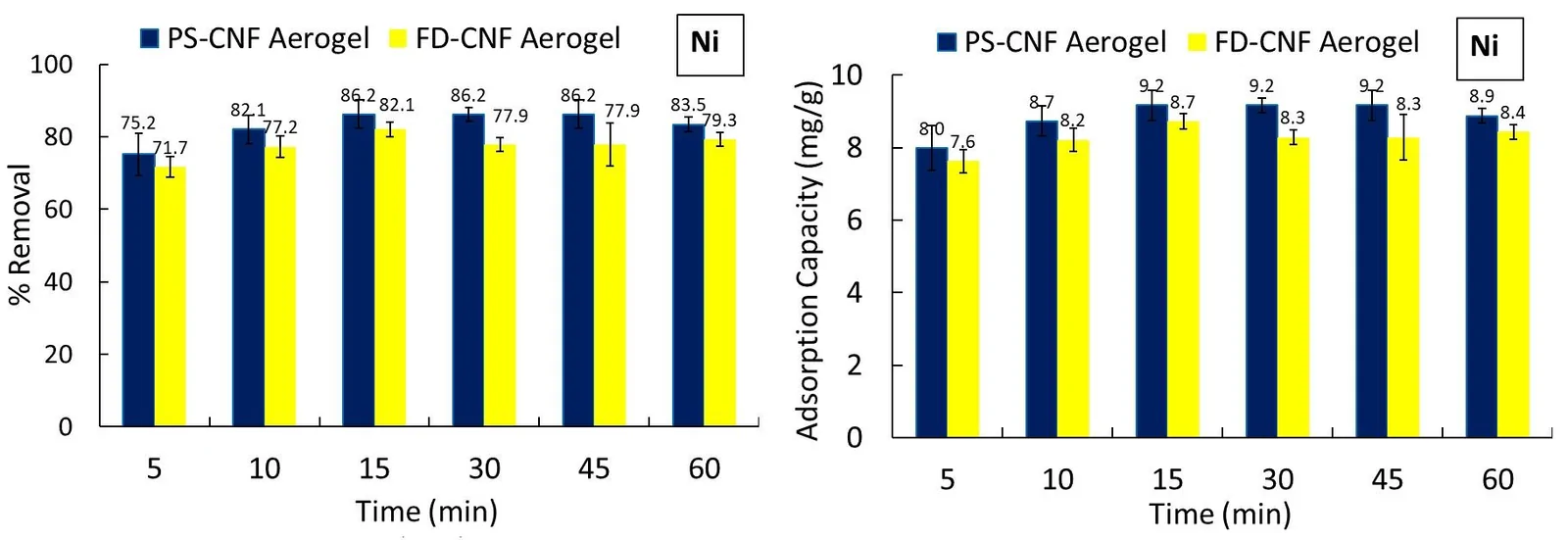

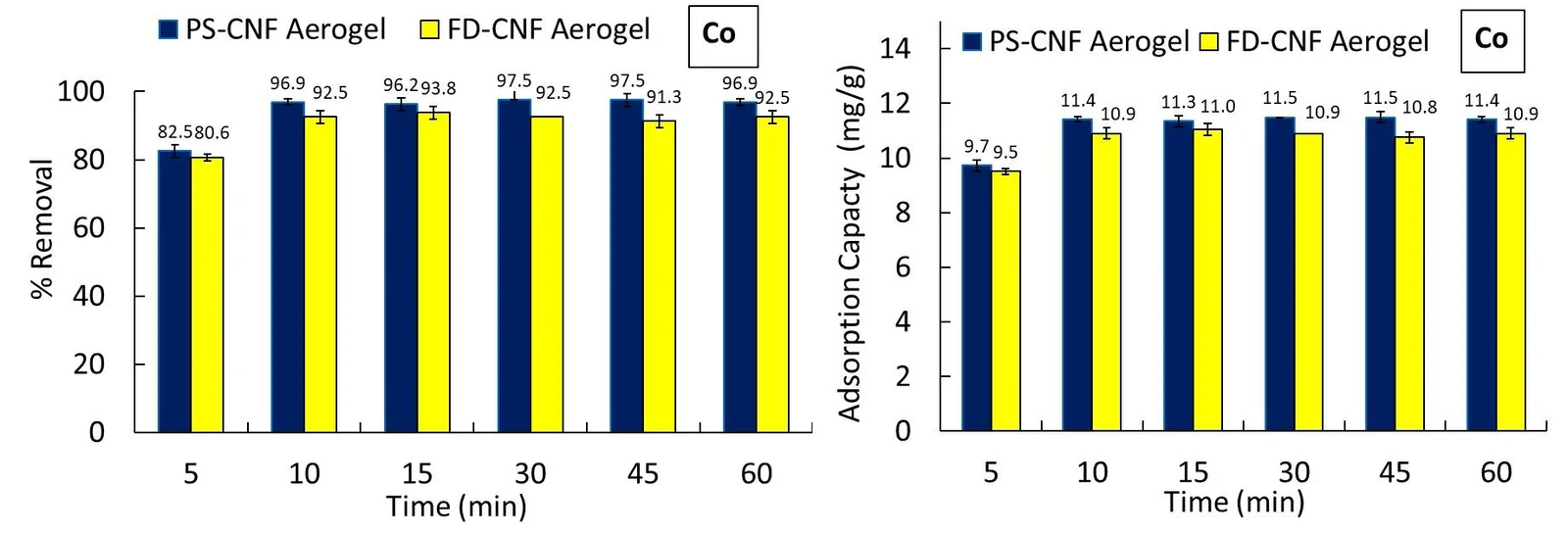

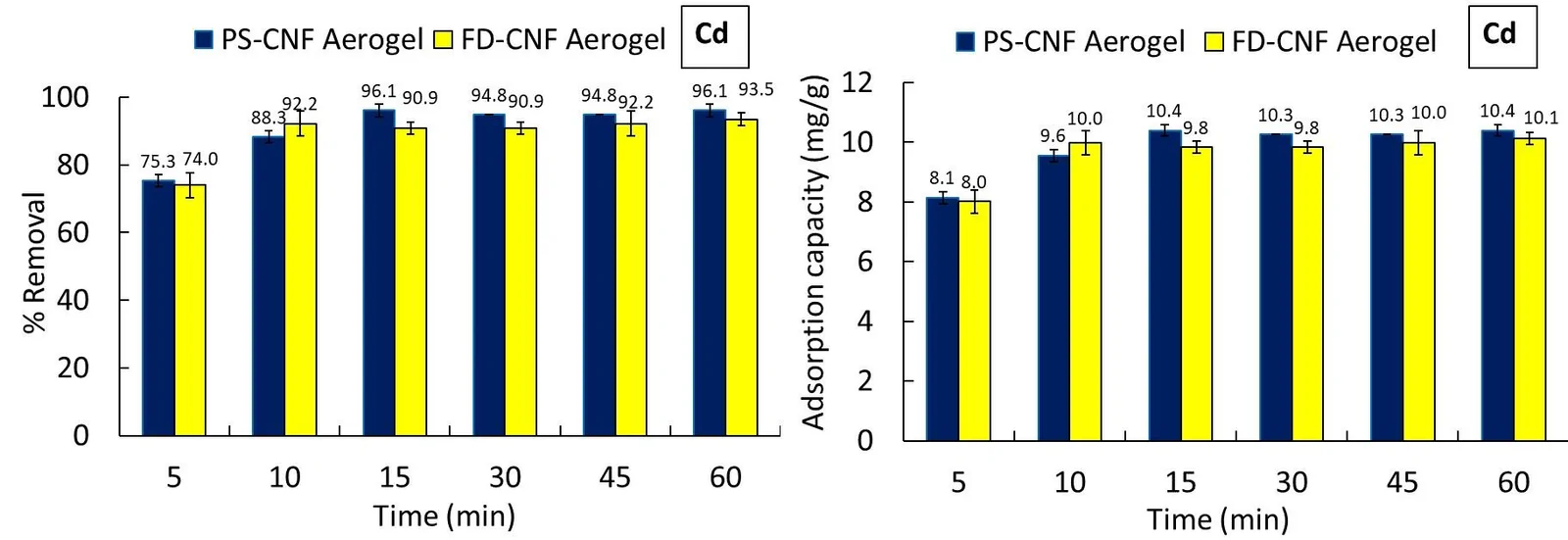

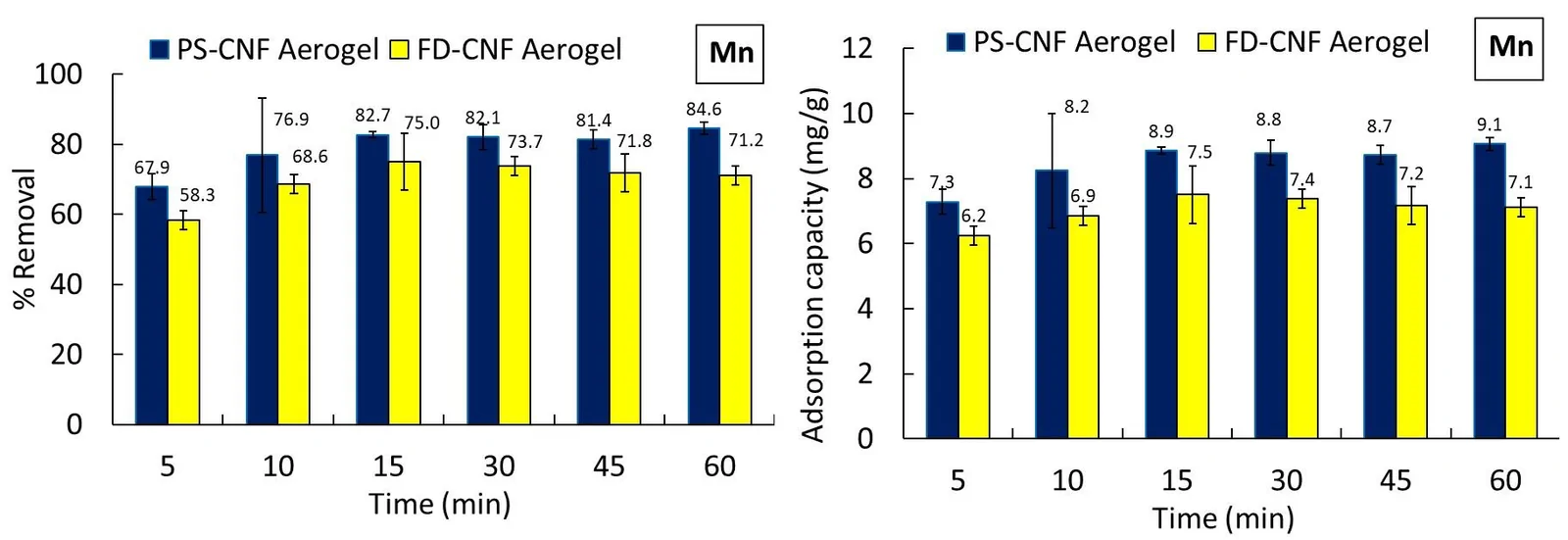

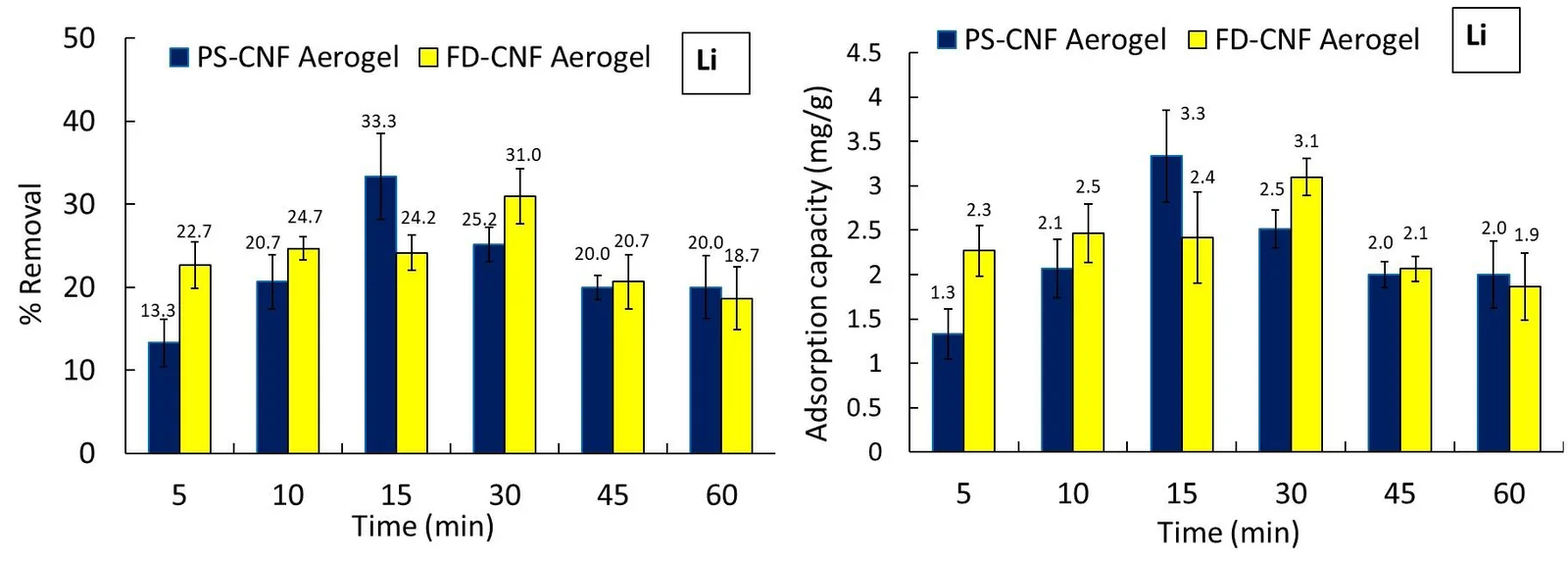
Effect of contact time on the adsorption of Pb, Ni, Co, Mn, Cd, and Li metal ions by PS-CNF and FD-CNF aerogels.

The effect of contact time on metal ion adsorption was evaluated at intervals of 5, 10, 15, 30, 45, and 60 min, with the initial metal ion concentration fixed at 100 ppm. Figure 8 illustrates the impact of contact time on the adsorption efficiency of the CNF aerogels. The results demonstrate rapid metal ion uptake by the different aerogel formulations, with equilibrium being attained within 15 to 30 min. Overall, the PS-CNF aerogels exhibit higher metal ion adsorption capacities than the FD-CNF aerogels, with the exception of Li ions. Furthermore, extending the contact time beyond 30 min for Li^+^ adsorption results in a decreased adsorption capacity. This kinetic desorption of Li^+^ is likely driven by a competitive back-exchange with Na^+^ ions released into the bulk solution during the initial uptake phase [70]. Because the TEMPO-CNF was prepared in an alkaline medium, Na^+^ ions originally occupied both the carboxylate (–COO^−^) groups and the hydroxyl (–OH) groups of the cellulose backbone. In contrast, the divalent heavy metal ions do not undergo this time-dependent desorption, as they shed their hydration shells to form strong, irreversible inner-sphere coordinate covalent bonds with the oxygen atoms of two adjacent –COO^−^ groups [70,71].

##### Kinetics of the Adsorption of Metal Ions by PS-CNF and FD-CNF

The time-dependent adsorption data in Figure 8 were fitted to pseudo-first-order (PFO) and pseudo-second-order (PSO) models to investigate the kinetics of the adsorption by PS-CNF and FD-CNF aerogels in order to determine the rate-limiting step of metal ions during the adsorption process and the mechanism of metal ions’ transport and binding onto the CNF aerogel surfaces [72].

Equations (5) and (6) describe the linear equations used for the PFO and PSO models, respectively [72].

(5) *ln*(*q_e_* − *q_t_*) = *ln*(*q_e_*) − *K*_1_*t*

(6) $$\frac{t}{q_{t}}=\frac{1}{K_{2}q_{e}^{2}}+\frac{1}{q_{e}}$$

where *q_t_* is the adsorption capacity at time *t* (mg/g), *q_e_* is the adsorption capacity at equilibrium (mg/g), *k*_1_ is the PFO rate constant (1/min), *k*_2_ is the PSO rate constant (g/(mg·min)), and *t* is the contact time (min).

The PFO model assumes that the adsorption rate is directly proportional to the number of unoccupied surface sites; this model typically points to physical adsorption (physisorption) driven by weak van der Waals forces and diffusion. On the other hand, the PSO model assumes that the adsorption rate is proportional to the square of the available active sites [73]. A strong statistical fit for the PSO model indicates that chemical adsorption (chemisorption) is the rate-limiting step, meaning the metal ions and the CNF aerogels are interacting through the direct sharing or exchanging of electrons [73].

The results in Table 6 show that the PSO model yielded significantly higher correlation coefficients (*R*^2^), indicating the rate-limiting step depends simultaneously on the metal ion concentration and the availability of unoccupied active sites in the case of PS-CNF and FD-CNF aerogels [73]. While PS-CNF aerogel demonstrated a slightly higher equilibrium capacity (q_e_), FD-CNF adsorbed these ions faster (higher K_2_ values). Both kinetic models failed to adequately describe Li ion adsorption, yielding physically impossible negative k_2_ values for the pseudo-second-order (PSO) model and poor correlation coefficients (R^2^) for the pseudo-first-order (PFO) model. This failure can be attributed to the complex binding mechanism of Li^+^ ions, which can interact with both the carboxylate and hydroxyl groups on the cellulose fibers, as well as the residual kaolin. The simultaneous involvement of these distinct binding sites creates a highly heterogeneous adsorption environment. More critically, at longer contact times (>15–30 min), the adsorption of Li ions tends to significantly decrease. Consequently, the system does not follow the uniform, single-site saturation kinetics or maximum adsorption capacity assumed by standard PFO and PSO models, resulting in complex, fluctuating mass transfer behavior rather than a stable equilibrium plateau [74]. The strong conformance to both the Langmuir isotherm and pseudo-second-order (PSO) kinetic models suggests that the adsorption process is strictly rate-limited by the localized interaction between the divalent metal ions and specific homogeneously distributed active sites in both of PS-CNF and FD-CNF—namely the carboxylate (COO^−^) and hydroxyl groups along the functionalized cellulose backbone [69,74] as well as the residual kaolin in the case of PS-CNF.

#### 2.4.3. Effect of Temperature on Metal Ion Adsorption

**Figure 9:**
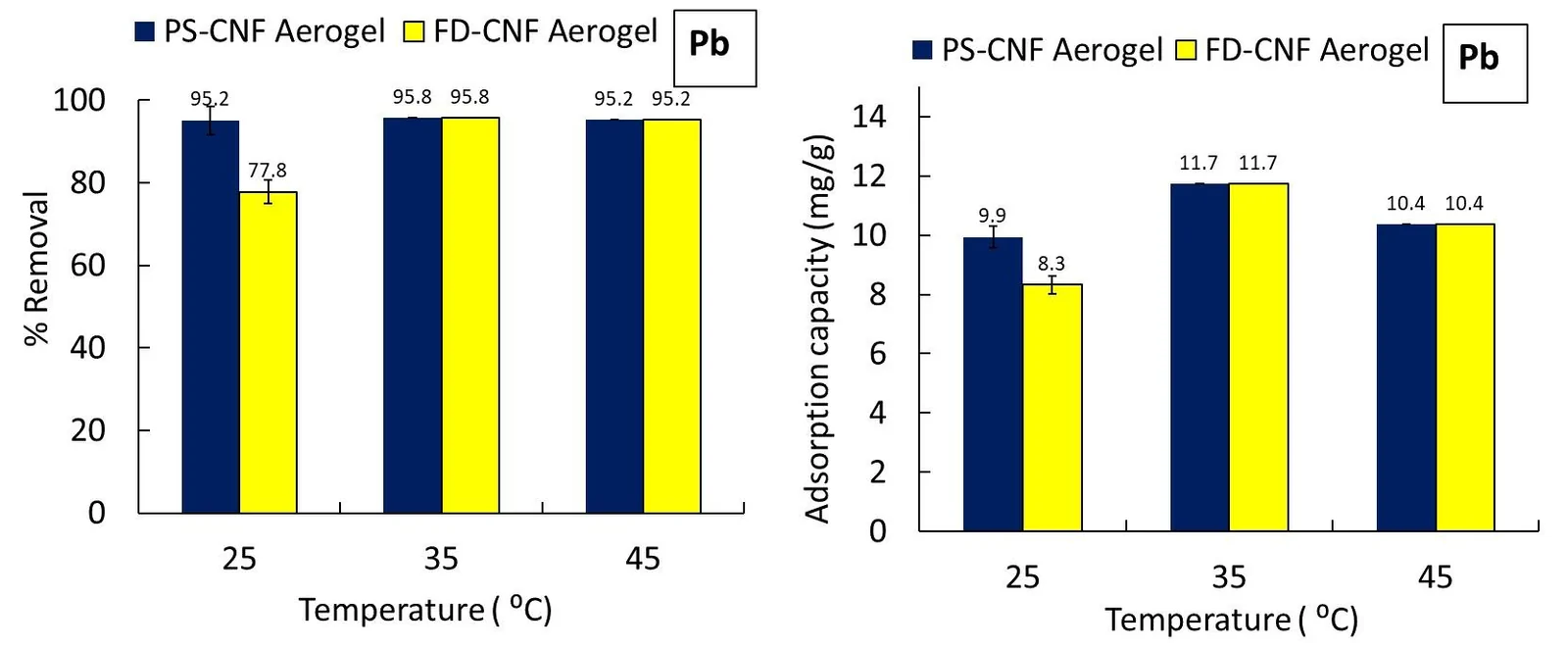

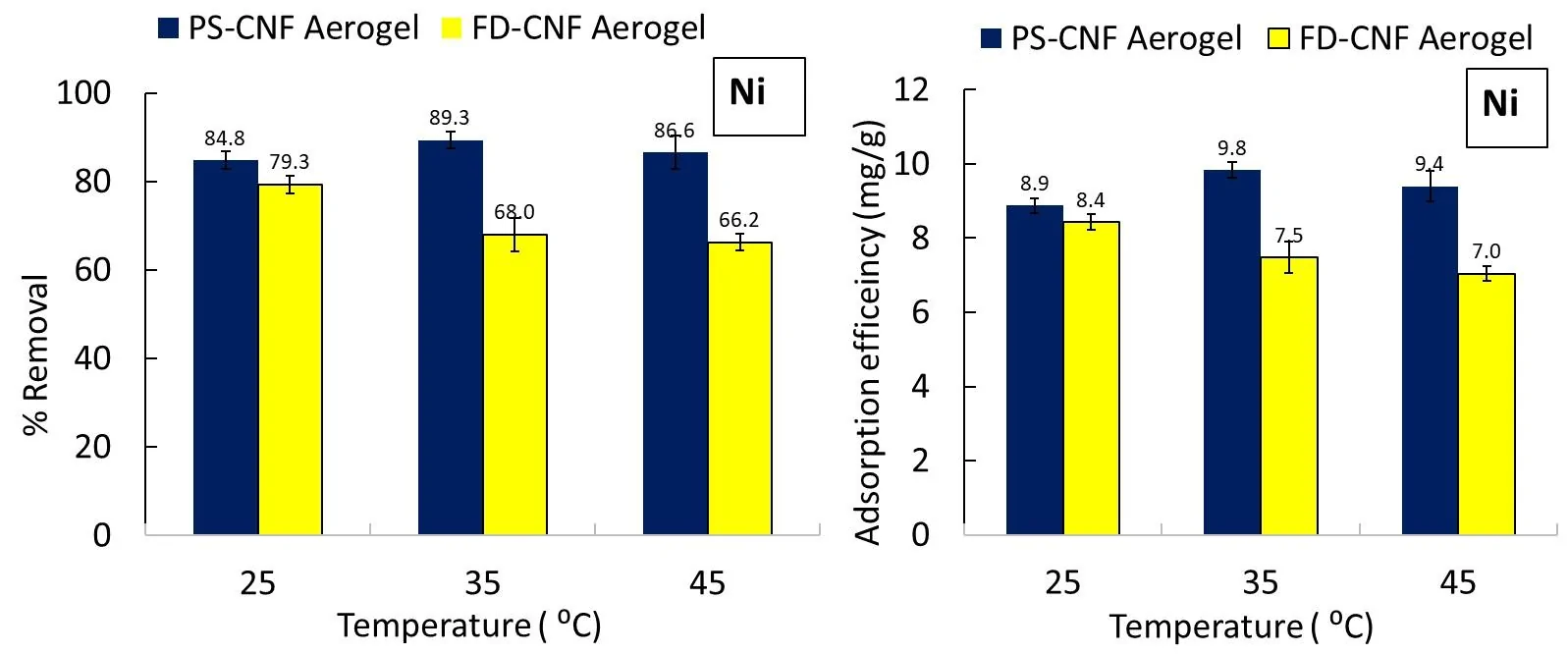

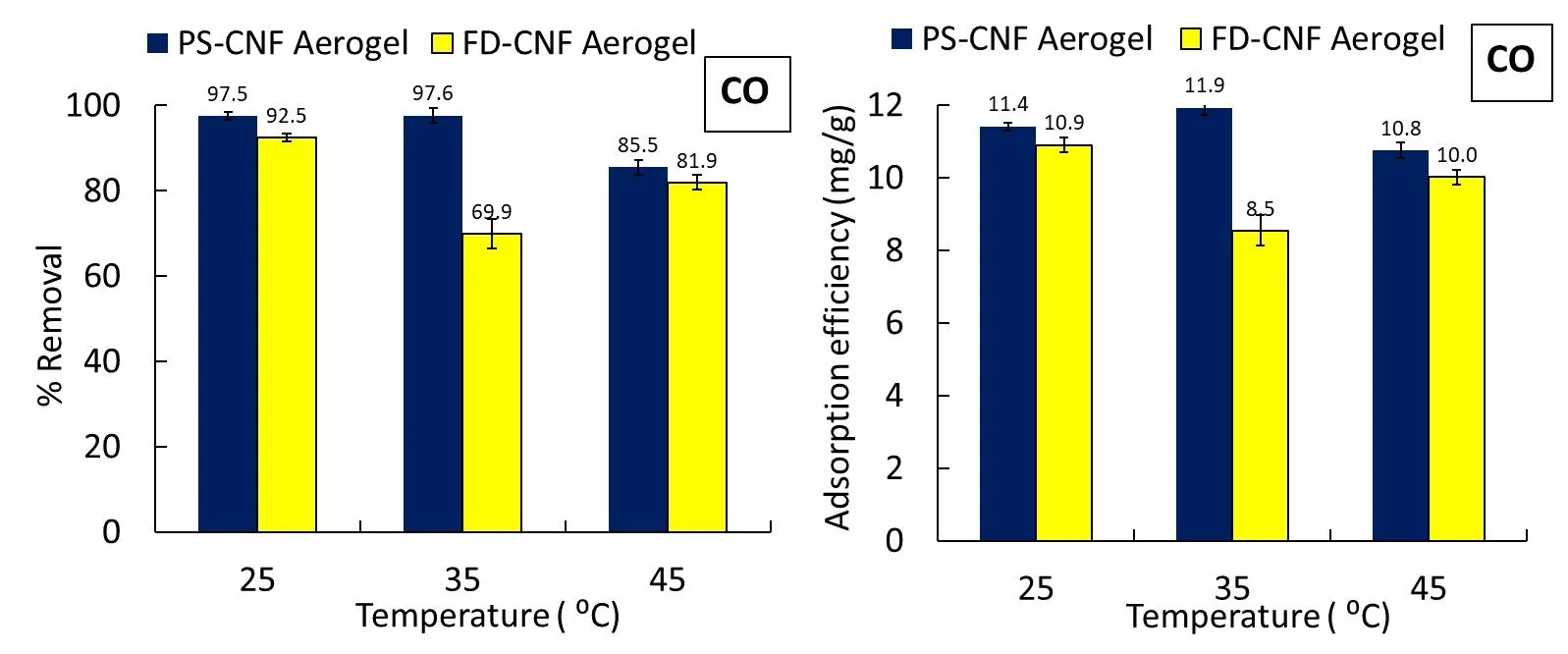

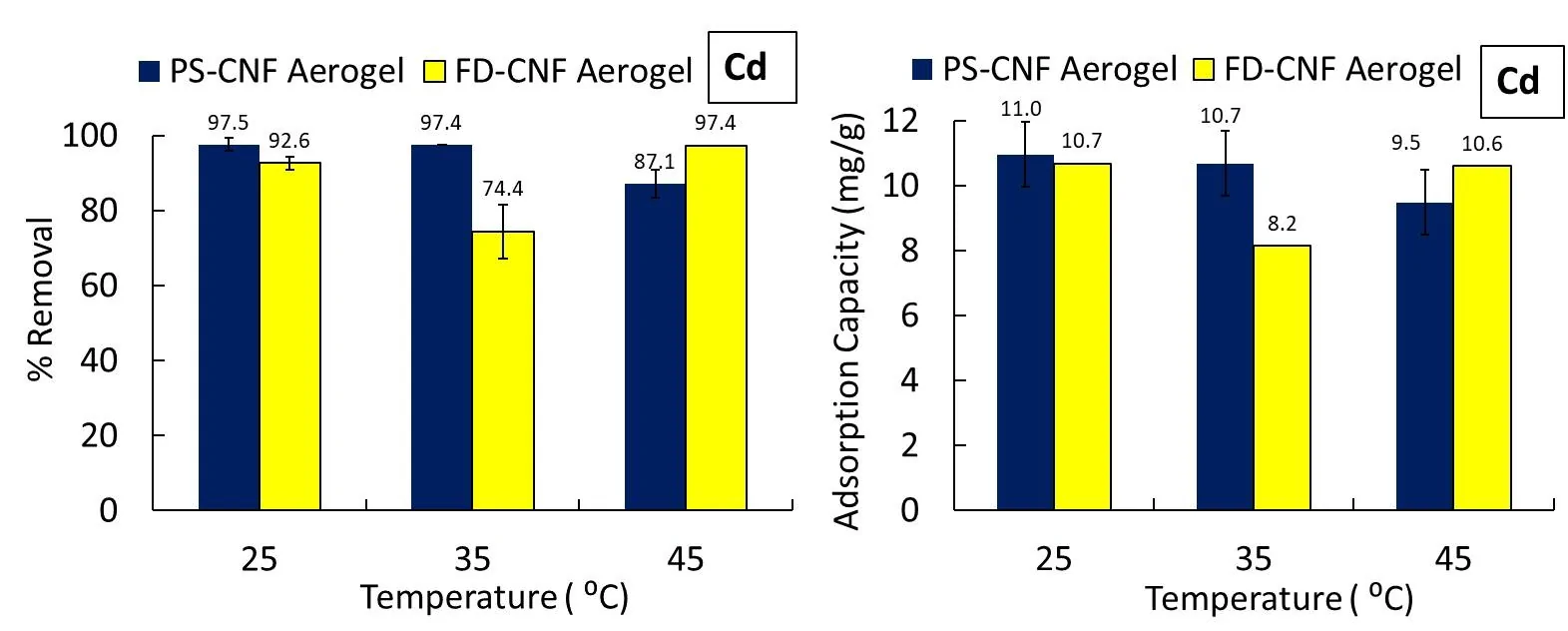

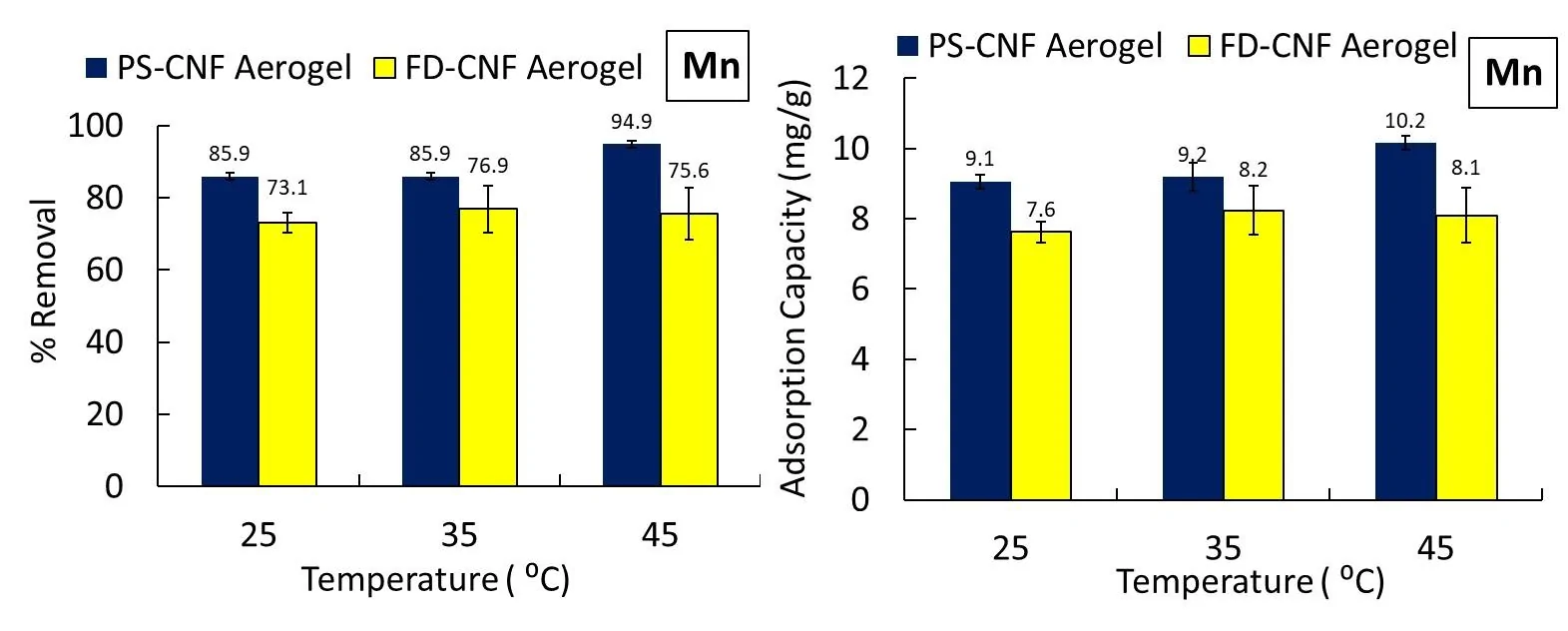

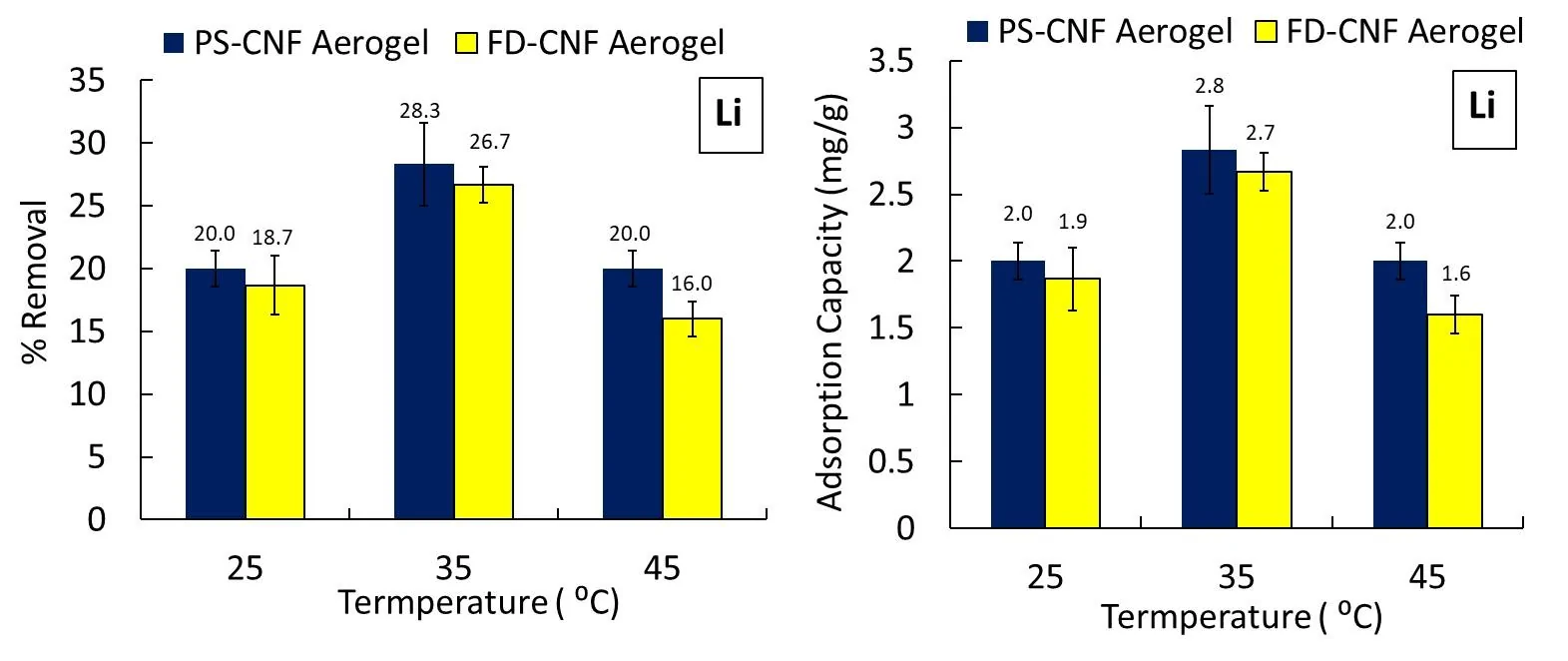
Effect of temperature on the adsorption of Pb, Ni, Co, Mn, Cd, and Li metal ions by PS-CNF and FD-CNF aerogels.

The effect of temperature on metal ion adsorption was investigated at 25 °C, 35 °C, and 45 °C using an initial metal concentration of approximately 100 mg/L and a contact time of 30 min (Figure 9). Overall, metal uptake by both PS-CNF and FD-CNF aerogels exhibited a distinctly non-linear temperature dependence [68,75]. For the majority of the tested cations, maximum adsorption efficiency was achieved within an optimal temperature of 25–35 °C, followed by a decrease at higher temperature (45 °C). This trend in the effect of temperature on metal ion adsorption has been reported before with different metal ions and adsorbents [75,76,77]. In the 25–35 °C range, moderate thermal energy supplies sufficient activation energy to facilitate partial desolvation and enhance metal ions’ diffusion into the aerogel matrix [52,76]. Above 35 °C, however, the increased thermal kinetic energy weakens surface coordination bonds and promotes ion detachment, shifting the equilibrium toward desorption. The decline in adsorption efficiency with increasing temperature is consistent with an exothermic adsorption process [76].

#### 2.4.4. Competitive Adsorption of Metal Ions

**Figure 10:**
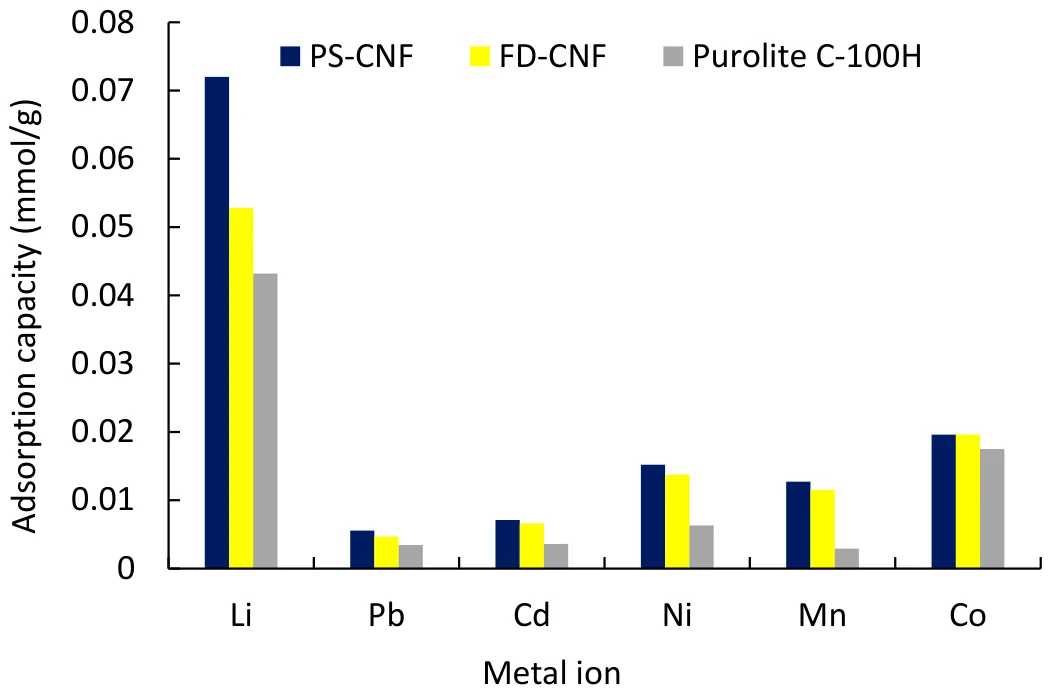
Differential adsorption of metal ions from a mixed solution by PS-CNF, FD-CNF, and Purolite C-101.

The competitive adsorption capacities of the prepared paper sludge (PS-CNF) and fiber dust (FD-CNF) aerogels for a mixture of the studied metal ions (Li, Pb, Cd, Ni, Mn, and Co) were evaluated and compared against a commercial cation exchange resin, Purolite C-100H (a strong cation-exchange resin with sulfonic acid groups). As the results in Figure 10 show, both CNF aerogels generally outperform the commercial resin across the multi-metal system.

The CNF aerogels showed higher efficiency than the commercial resin despite the higher adsorption capacity stated by the manufacturer (total volume capacity 2 meq/l or as mass capacity 2.44 meq/g), which could be attributed to the difference in the form and density, i.e., porosity and microstructure. The CNF aerogels showed significantly larger porosity than the commercial resin beads (~0.3–1 mm diameter); the bulk specific gravity of the CNF aerogels ranged from 0.0490 to 0.0338 g/cm^3^, while that of the resin beads was 1.20 g/cm^3^. The porosity of the aerogels was ~97%, while the estimated porosity of the commercial resin (based on its moisture retention stated by the manufacturer) is 55–60%. The surface area of the aerogel wall layers is also high (~270 m^2^/g), significantly higher than that of the commercial resin (~20–45 g/m^2^). Furthermore, the CNF aerogel has higher wettability and water adsorption than the commercial resin, as the aerogel consists of a highly hydrophilic polysaccharide skeleton with abundant hydroxy and carboxylic groups, while the commercial resin has a hydrophobic polystyrene–divinyl benzene backbone. All the aforementioned factors contribute to the higher metal ion adsorption capacity of the CNF aerogel when compared to the commercial resin.

Notably, the PS-CNF aerogel exhibited a higher adsorption capacity than the FD-CNF aerogel, a result that is in agreement with previous single-metal ion adsorption tests. The distinct adsorption hierarchy observed (Li ≫ Co > Ni > Mn > Cd > Pb) can be interpreted based on the higher availability of Li ions due to the higher concentration of Li than other metal ions in the mixture; other factors—including the steric confinement and ionic dimensions and specific coordination chemistry within the three-dimensional CNF aerogels—should be taken into consideration.

The adsorption order is influenced by the intrinsic bare ionic radii of the metals [78]. Large ions like Pb^2+^ (1.19 Å) and Cd^2+^ (0.95 Å) experience severe steric hindrance, restricting them from diffusing deeply into the tight internal pores of the highly crosslinked aerogels in order to reach active binding sites [78]. Conversely, much smaller ions like Li^+^ (0.76 Å) and Co^2+^ (0.745 Å) can easily penetrate the matrix. These smaller ions shed their hydration shells to form strong complexes directly with the dense network of oxygen-rich functional groups on the cellulose backbone [79]. Consequently, both PS-CNF and FD-CNF exhibit exceptional high adsorption for Li^+^, bypassing the bulkier multivalent ions entirely. Li^+^ ions can easily react with both the COO- and OH groups of CNF as compared to the other divalent cations [79]. Although Ni^2+^ (0.69 Å) has a slightly smaller bare radius than Co^2+^, the aerogels exhibit a preferential uptake for cobalt. This is driven by specific coordination chemistry, where functional groups such as carboxyl and hydroxyls on the adsorbent surface enhance the chemisorption of specific transition metals like Co^2+^ over competitors [80].

To estimate the selectivity of the metal ions regardless their concentration in the solution mixture, the distribution coefficient (*K_D_*) of adsorption for each metal ion was calculated and the results are presented in Table 7; using the *K_D_* as a metric normalizes the binding affinity against the differing starting concentrations and describes the affinity of an adsorbate for a specific adsorbent amongst several competing ions [81]. The distribution coefficient (*K_D_*) was calculated as follows [81,82]:

$$K_{D}=\frac{{(C}_{0}-C_{e})\times V}{C_{e}\times m}$$

where *C*_0_ is the concentration of the metal ion in the solution mixture before adsorption, *C_e_* is the equilibrium concentration (mmol/g), *V* is the volume of solution (L), and *m* is the mass of adsorbent used for the adsorption.

As evidenced by the calculated K_D_ values, the aerogels exhibit a distinct selectivity sequence, preferentially binding specific heavy and transition metals over others. For the more efficient PS-CNF aerogel, the specific selectivity order is Co > Mn > Ni ≈ Cd > Pb > Li. While lithium consistently exhibited the lowest binding affinity across all materials (ranging from 34.6 to 75.0 mL/g), the aerogels demonstrated a very high, targeted selectivity for cobalt (8650.0 mL/g for both aerogels) and manganese (2100.0 mL/g for PS-CNF). This pronounced difference in selectivity among the heavy metals indicates that the binding sites possess a specific thermodynamic preference, likely governed by the distinct electronegativity, hydrated radii, and Lewis acid behavior of each respective metal ion [81,83]. Furthermore, the PS-CNF aerogel consistently outperformed both the FD-CNF aerogel and the commercial Purolite C-100H resin across this selectivity spectrum. Specifically, PS-CNF exhibited K_D_ values roughly double to triple those of the FD-CNF aerogel for Pb, Cd, Ni, and Mn and vastly exceeded the commercial resin (e.g., demonstrating a 77-fold higher affinity for Mn). This enhanced capacity and distinct selectivity profile further corroborate the added value of the residual kaolin naturally present within the PS-CNF matrix, which not only provides additional high-affinity binding sites for targeted heavy metal capture but also contributes to the formation of an expanded, multi-layered aerogel structure, as observed in the SEM images.

## 3. Conclusions

This study successfully demonstrates the viability of utilizing primary paper sludge (generated from the recycling of writing and printing paper) and fiber dust waste (generated during tissue paper trimming) to fabricate highly porous CNF aerogels for the remediation of battery-related metal ions from aqueous environments. Through a comparative analysis of the PS-CNF and FD-CNF aerogels under the evaluated conditions, it can be concluded that the presence of residual kaolin (~12%) within the fibrils of the PS-CNF acts as a physical spacer during freeze-drying. This prevents fibril agglomeration, resulting in an expanded macroporous wall architecture with a high specific surface area (~271 m^2^/g) and yielding a higher compressive strength compared to the FD-CNF aerogel made from pure cellulose pulp.

When tested for the removal of Pb^2+^, Ni^2+^, Co^2+^, Cd^2+^, Mn^2+^, and Li^+^ ions, the PS-CNF aerogel generally outperformed the FD-CNF despite the latter’s slightly higher carboxylic content, an enhancement that can be attributed to the residual kaolin and the less dense aerogel microscopic structure of the PS-CNF aerogel. Maximum experimental capacities of 39.28 mg/g for Pb^2+^, 29.80 mg/g for Cd^2+^, 21.05 mg/g for Co^2+^, 20.19 mg/g for Mn^2+^, 16.80 mg/g for Ni^2+^, and 11.33 mg/g for Li^+^ were achieved using the PS-CNF aerogel. Metal uptake by both aerogels was rapid and predominantly governed by chemisorption on a homogeneous surface, as evidenced by strong fits to the pseudo-second-order kinetic and Langmuir isotherm models. Notable exceptions included Pb^2+^, which followed Freundlich behavior due to steric hindrance from its larger ionic radius, and Li^+^, which exhibited complex, multi-site binding interactions that deviated from standard equilibrium models. Optimal adsorption efficiencies were achieved at moderate temperatures between 25 °C and 35 °C, before declining at higher temperatures.

In competitive multi-metal solutions, both the PS-CNF and FD-CNF aerogels outperformed a commercial Purolite C-100 cation exchange resin. The PS-CNF aerogel demonstrated the highest overall efficiency and exhibited a strong selective affinity for the transition and heavy metal ions, particularly Mn^2+^ and Co^2+^, over the monovalent Li ions.

Ultimately, this study proves the potential of valorizing a negative-value waste—primary paper sludge from recycling and de-inking—to engineer low-cost adsorbents (as compared with virgin cellulose pulp) for the recovery of critical metal ions for the battery industry. The presence of kaolin in this sludge is an asset rather than a limitation; its inclusion reduces the need for exhaustive purification while simultaneously enhancing the structural integrity and heavy metal adsorption capacity of the nanocellulose aerogels, presenting a potentially highly sustainable, cost-effective strategy for treating toxic effluents.

While this study relies on established isotherm and kinetic model fitting (R^2^) to evaluate adsorption capacity, future studies would benefit from advanced statistical treatments, such as rank aggregation and pairwise comparisons [84], to further validate comparative sample performance.

## 4. Materials and Methods

### 4.1. Materials

Primary paper sludge from recycled printing and writing paper (~35% solid content) and fiber dust (6.8% moisture content) were generously provided by Interstate Company for Paper Products (Sadat City, Egypt). Reagent-grade 2,2,6,6-tetramethylpiperidine-1-oxyl), sodium bromide, and a 14% sodium hypochlorite solution were acquired from Sigma-Aldrich (St. Louis, MO, USA). A commercial polyamidoamine–epichlorohydrin (PAE) wet-strength additive (~27% aqueous solution) was obtained from Solenis LLC (Wilmington, DE, USA). Purolite C-100H cation exchange resin was sourced from Ecolab Bioprocessing and Purification Technologies (King of Prussia, PA, USA). All metal precursor salts—lead(II) chloride, cadmium chloride, manganese(II) chloride tetrahydrate, cobalt(II) chloride hexahydrate, nickel(II) chloride hexahydrate, and lithium chloride—were of reagent grade, purchased from Thermo Fisher Scientific (Waltham, MA, USA), and used without further purification.

### 4.2. Pretreatment of Paper Sludge

To remove the acid-soluble ash content (predominantly calcium carbonate), the paper sludge was de-ashed using formic acid following a previously reported method [34]. Briefly, the sludge was reacted with 0.01 N formic acid at a solid-to-liquid ratio of 1:10 for 1 h at 40 °C under continuous stirring. After the acid treatment, the de-ashed sludge was washed thoroughly with distilled water until neutral pH was achieved, mechanically pressed to ~30% solid content, and refrigerated at 4 °C until subsequent nanofiber isolation.

### 4.3. Preparation of CNF from Paper Sludge and Fiber Dust

For CNF isolation, both the de-ashed paper sludge and fiber dust were subjected to TEMPO-mediated oxidation following a previously reported method [85]. Briefly, 30 g (dry weight) of the cellulosic material was suspended in a 2 L aqueous solution containing 0.48 g of TEMPO and 4.8 g of sodium bromide. The reaction pH was adjusted and maintained at 10.0 by dropwise addition of sodium hypochlorite solution. After reacting for 2 h at room temperature, the oxidation was quenched by adding 20 mL of ethanol. The suspension was centrifuged at 10,000 rpm using a Sigma 1-13K centrifuge (Sigma Laborzentrifugen GmbH, Osterode am Harz, Germany) to remove the supernatant, followed by repeated washing cycles with distilled water until neutral pH was reached. The oxidized fibers were subsequently dialyzed against distilled water for 48 h using cellulose dialysis tubing (MWCO 14,000 Da; Sigma-Aldrich, St. Louis, MO, USA). To individualize the nanofibers, the oxidized fiber suspension (~4% consistency) was sonicated for 5 min in an ice bath using a UP400 ultrasonic processor (Hielscher Ultrasonics GmbH, Teltow, Germany) equipped with a 22 mm diameter probe at 70% power. The resulting CNF suspensions were stored at 4 °C until further use.

### 4.4. Characterization of Cellulose Nanofibers

#### 4.4.1. Transmission Electron Microscopy (TEM)

TEM was carried out using a JEOL high-resolution transmission electron microscope (HR-TEM, JEM-2100, JEOL, Tokyo, Japan). Phosphotungstic acid staining was applied before examination.

#### 4.4.2. Carboxylic Acid Content Determination

The carboxyl group content of the nanofibers was quantified via conductometric acid–base titration according to a previously reported protocol [85]. Briefly, 100 mg (dry basis) of CNF was suspended in 10 mL of 0.01 M HCl and stirred for 10 min to ensure complete protonation of all carboxylate moieties. The resulting mixture was subsequently titrated against standardized 0.005 M NaOH under continuous stirring. Real-time conductivity changes throughout the titration were recorded using a Jenway 7200 conductivity meter (Jenway, Stone, Staffordshire, UK). The carboxyl content in mmol/g was derived from the neutralization plateau of the conductometric curves using the following equation:

(7) $$X=\frac{\left(V_{2}-V_{1}\right)\times C}{m}$$

where *V*_1_ is the volume of NaOH consumed to neutralize the excess strong acid (the first inflection point), *V*_2_ is the cumulative volume of NaOH required to neutralize both the strong acid and the carboxyl groups (the second inflection point), *C* is the molar concentration of the NaOH titrant, and *m* is the dry mass of the CNF sample (g).

#### 4.4.3. Zeta Potential Measurement

The surface charge of the isolated CNFs was determined via zeta potential (ζ) analysis using a Zetasizer Nano ZS (Malvern Panalytical, Malvern, UK; 633 nm He–Ne laser) at 25 °C. Aqueous suspensions (0.2 wt%) were equilibrated for 10 min in quartz cuvettes prior to measurement. Electrophoretic mobilities were recorded in automatic mode (up to 1000 acquisitions) and converted to ζ-potentials using the Smoluchowski approximation (Henry’s F(κa) = 1.5).

### 4.5. Preparation of CNF Aerogel

To prepare the aerogels, PAE (4 wt% relative to dry CNF) was thoroughly blended into the nanofiber suspension as a crosslinking agent. The formulated mixture was frozen at −12 °C for 12 h and subsequently lyophilized for 48 h using an Alpha LSCbasic freeze-dryer (Martin Christ Gefriertrocknungsanlagen GmbH, Osterode am Harz, Germany). Covalent crosslinking was subsequently completed by thermally curing the dry aerogels in a forced-air convection oven at 80 °C for 15 min.

### 4.6. Characterization of CNF Aerogel

#### 4.6.1. SEM/EDX Analysis

The surface morphology and elemental composition of the aerogels were examined using a Quanta 200 scanning electron microscope coupled with an energy-dispersive X-ray spectrometer (SEM/EDX; FEI Company BV, Eindhoven, The Netherlands) operated at an accelerating voltage of 20 kV. Prior to examination, the samples were sputter-coated with an ultrathin layer of gold using an Edwards sputter coater (Edwards, Burgess Hill, UK) to prevent surface charge accumulation during analysis.

#### 4.6.2. XRD Analysis

The crystalline structure of the CNF aerogels was analyzed via XRD using a Bruker D8 Advance diffractometer (Bruker AXS, Karlsruhe, Germany) equipped with monochromatic Cu K_α_ radiation (λ = 1.5406^0^) operated at 40 kV and 40 mA. Diffraction profiles were recorded at 25 °C over a 2-θ scanning range of 10–80° with an angular step size of 0.02° and a scan speed of 4 s per step. The relative crystallinity index was calculated using the following equation [86]:

(8) $$\;\mathrm{Crystallinity}\;(\%)=\;\frac{I_{200}-I_{\mathrm{am}}}{I_{002}}\times 100$$

where *I*_200_ is the diffractogram height of (200) peak, and *I_am_* is the height of the amorphous background at 2θ ~ 19.

#### 4.6.3. Wavelength-Dispersive X-Ray Fluorescence (XRF) Analysis of Ash in Paper Sludge

XRF analysis was carried out using an Axios advanced sequential WDXRF spectrometer (Panalytical, Malvern, UK).

#### 4.6.4. Bulk Density and Porosity

The bulk density (*ρ*) of the prepared aerogels was calculated according to the following equation:

(9) $$\rho_{b}=\frac{\mathrm{Mass}\;\left(g\right)}{\mathrm{volume}\;\left({\mathrm{cm}}^{3}\right)}$$

The porosity (*P*) was calculated using the following equation:

(10) $$P\;\left(\%\right)=\left(1-\;\frac{\rho_{b}}{\rho_{s}}\right)\;100$$

where *ρ_b_* is the bulk density and *ρ_s_* is the skeletal density of cellulose (1.5 g/cm^3^).

#### 4.6.5. Compression Strength Testing

The compressive strength of the aerogels was measured using an LR-10X Lloyd universal testing machine (Lloyd Instruments Ltd., Bognor Regis, UK) operating at a crosshead speed of 2 mm/min at 25 °C. Five replicate specimens were tested for each aerogel sample, and the average values are reported.

#### 4.6.6. BET Surface Area Measurement

The specific surface area, pore size distribution, and total pore volume of the prepared aerogels were determined from the nitrogen adsorption–desorption isotherms using the BET method and measured by a NOVA Touch 4LX surface area and porosity analyzer (Quantachrome Instruments, Boynton Beach, FL, USA) at 77.4 K. The samples were degassed at 373 K for 4 h under a vacuum to remove adsorbed moisture or volatile impurities.

### 4.7. Metal Ion Adsorption Tests

#### 4.7.1. Single-Metal Ion Adsorption

Single-component adsorption tests for Ni^2+^, Co^2+^, Mn^2+^, Pb^2+^, Cd^2+^, and Li^+^ by the PS-CNF and FD-CNF aerogels were conducted in batch mode. The suspensions were agitated at 150 rpm in a temperature-controlled water bath. For each test, 0.05 g of the respective aerogel was added to 5 mL of the metal ion solution. To evaluate the effect of initial concentration, metal solutions of 50, 100, 150, 200, 250, and 500 mg/L were utilized. To assess the impact of contact time, adsorption was monitored at intervals of 5, 15, 30, 45, and 60 min using an initial concentration of 100 mg/L at 25 °C. Thermodynamic temperature effects were evaluated by performing tests at 25, 35, and 45 °C. The residual concentrations of the divalent heavy metals (Ni^2+^, Co^2+^, Mn^2+^, Pb^2+^, and Cd^2+^) were determined via standard complexometric EDTA titration [87], whereas the Li^+^ concentration was quantified using an Agilent 7500CE inductively coupled plasma–mass spectrometer (ICP-MS; Agilent Technologies, Inc., Santa Clara, CA, USA). All experiments were performed in duplicate, and the results were averaged.

#### 4.7.2. Mixed-Metal Ion Adsorption

For competitive adsorption studies, a multi-metal mixture was prepared by combining 5 mL of each 100 mg/L single-ion stock solution. The batch test was conducted using 0.05 g of aerogel or the Purolite C100 cation exchanger and 5 mL of this mixed solution. Following the adsorption period of 30 min at 25 °C, the suspension was separated as previously described. The concentrations of all metal ions in the mixture, both before and after adsorption, were measured using the Agilent 7500CE ICP-MS instrument. These tests were run in duplicate and the results averaged.

## Figures and Tables

**Table 1:** Surface area, total pore volume, and average pore size of PS-CNF and FD-CNF aerogels.

Aerogel Type	BET Surface Area (m^2^/g)	Total Pore Volume (cm^3^)	Average Pore Size (nm)
FD-CNF	269.4	0.1404	1.043
PS-CNF	271.1	0.1514	1.117

**Table 2:** XRF analysis for the main elements of paper sludge after dilute formic acid pretreatment.

Element	Constituent	Wt. %
Ca	CaO	0.41
Si	SiO_2_	5.99
Al	Al_2_O_3_	3.25
Fe	Fe_2_O_3_	0.87
Mg	MgO	0.40
S	SO_3_	0.25
Ti	TiO_2_	0.22
Cl	Cl	0.13
Cu	CuO	0.099
K	K_2_O	0.08
P	P_2_O_5_	0.06
Zn	ZnO	0.022
Co	Co_3_O_4_	0.016
Sr	SrO	0.011
Zr	ZrO_2_	0.028
Ni	NiO	0.01
Mn	MnO	0.009
Cr	Cr_2_O_3_	0.009
Pb	PbO	0.005
Loss due to ignition		88.13

**Table 3:** pH values of the prepared metal ion solutions.

Metal Ion Solution	pH of 50 ppm–500 ppm Solutions
Ni	6.65–6.52
Co	6.48–6.56
Cd	6.29–6.41
Pb	4.53–4.77
Mn	6.47–6.69
Li	7.43–7.48

**Table 4:** Experimental adsorption capacities of PS-CNF and FD-CNF aerogels for the different metal ions.

	Adsorption of Metal Ions by CNF Aerogels	
Metal Ion	PS-CNF in mg/g (mmol/g)	FD-CNF in mg/g (mmol/g)
Pb	39.28 (0.19)	37.91 (0.183)
Ni	16.8 (0.286)	11.81 (0.201)
Co	21.05 (0.357)	14.07 (0.239)
Cd	29.8 (0.265)	27.26 (0.242)
Mn	20.19 (0.368)	18.34 (0.334)
Li	11.33 (1.633)	10.53 (1.517)

**Table 5:** Parameters of the linear Langmuir, Freundlich, and Dubinin–Radushkevich isotherms for the different metal ion adsorption by PS-CNF and FD-CNF aerogels.

		Ni		Co		Cd		Mn		Pb		Li	
Model	Parameter	(PS-CNF)	(FD-CNF)	(PS-CNF)	(FD-CNF)	(PS-CNF)	(FD-CNF)	(PS-CNF)	(FD-CNF)	(PS-CNF)	(FD-CNF)	(PS-CNF)	(FD-CNF)
Langmuir	*q_m_* (mg/g)	17.21	12.17	21.33	14.16	30.21	28.08	21.94	21.84	53.08	65.72	6.81	10.09
	*K_L_* (L/mg)	0.09	0.09	0.11	0.64	0.04	0.06	0.03	0.01	0.029	0.006	0.014	0.005
	*R_L_*	0.02–0.52	0.02–0.54	0.02–0.16	0.03–0.14	0.02–0.14	0.03–0.26	0.06–0.35	0.12–0.57	0.08–0.475	0.07–0.253	0.13–0.60	0.29–0.8
	*R* ^2^	0.99	0.99	0.99	0.99	0.99	0.99	0.99	0.98	0.94	0.96	0.58	0.80
Freundlich	1/*n*	0.25	0.21	0.13	0.17	0.19	0.3	0.37	0.47	0.51	0.889	0.69	0.84
	*K_F_*	4.74	3.82	9.91	6.1	10.51	5.48	2.79	1.40	3.54	0.452	0.16	0.07
	*R* ^2^	0.87	0.89	0.96	0.81	0.76	0.84	0.9	0.92	0.99	0.96	0.83	0.92
Dubinin-Radushkevich	*q_s_* (mg/g)	13.58	10.43	17.52	13.39	24.39	19.01	14.69	12.63	25.45	24.06	6.50	6.36
	*K_DR_* (mol^2^/K_J__2_)	1.15	4.07	1.23	0.53	3.01	2.3	12.82	40.03	11.63	11.54	158.5	275.5
	*E* (KJ/mol)	0.66	0.35	0.64	0.97	0.41	0.47	0.2	0.11	0.21	0.07	0.06	0.04
	*R* ^2^	0.78	0.79	0.76	0.94	0.85	0.84	0.87	0.81	0.69	0.67	0.66	0.75

**Table 6:** Pseudo-first-order (PFO) and pseudo-second-order (PSO) kinetic parameters for the adsorption of the different metal ions by PS-CNF and FD-CNF aerogels.

		Ni		Co		Cd		Mn		Pb		Li	
		PS-CNF	FD-CNF	PS-CNF	FD-CNF	PS-CNF	FD-CNF	PS-CNF	FD-CNF	PS-CNF	FD-CNF	PS-CNF	FD-CNF
Pseudo-First-Order Kinetic Model	q_e_ (mg/g)	8.93	8.40	11.48	10.93	10.31	10.01	8.82	7.28	7.93	6.81	2.44	2.459
	K_1_	0.446	0.538	0.381	0.413	0.305	0.333	0.337	0.321	0.213	0.332	0.216	0.528
	R^2^	0.39	0.464	0.978	0.955	0.967	0.946	0.923	0.922	0.749	0.714	0.36	0.023
Pseudo-Second-Order Kinetic Model	q_e_ (mg/g)	9.08	8.41	11.55	10.92	10.55	10.22	9.13	7.21	8.55	6.92	1.995	1.864
	K_2_	0.283	0.828	0.22	0.397	0.098	0.114	0.102	0.556	0.0439	0.222	−0.257	−0.129
	R^2^	0.998	0.995	0.99	0.99	0.99	0.99	0.99	0.99	0.998	0.992	0.968	0.957

**Table 7:** Distribution coefficients (K_D_) of various metal ions for PS-CNF, FD-CNF, and Purolite C-100H in a competitive multi-component system.

	Distribution Coefficient (K_D_, mL/g)		
Metal Ion	PS-CNF	FD-CNF	Purolite C-100H
Li	75.0	45.8	34.6
Pb	627.3	263.6	115.1
Cd	1200.0	622.2	85.7
Ni	1218.2	504.2	61.1
Mn	2100.0	633.3	27.2
Co	8650.0	8650.0	775.0

## Data Availability

All data generated or analyzed during this study are included in this published article (and its Supplementary Information Files).

## Supplementary Materials

The following supporting information can be downloaded at https://www.mdpi.com/article/10.3390/gels12100935/s1, Figure S1: Surface area BET curves, total pore volume, and average pore radium of the PS-CNF aerogel, and Figure S2: Surface area BET curves, total pore volume, and average pore radium of the FD-CNF aerogel.

## References

[1] Brown C.W., Goldfine C.E., Allan-Blitz L.-T., Erickson T.B. (2024). Occupational, Environmental, and Toxicological Health Risks of Mining Metals for Lithium-Ion Batteries: A Narrative Review of the Pubmed Database. J. Occup. Med. Toxicol..

[2] Dagdag O., Quadri T.W., Haldhar R., Kim S.-C., Daoudi W., Berdimurodov E., Akpan E.D., Ebenso E.E. (2023). An Overview of Heavy Metal Pollution and Control. Heavy Metals in the Environment: Management Strategies for Global Pollution.

[3] Baird-Gunning J., Lea-Henry T., Hoegberg L.C.G., Gosselin S., Roberts D.M. (2017). Lithium Poisoning. J. Intensive Care Med..

[4] Crossgrove J., Zheng W. (2004). Manganese Toxicity upon Overexposure. NMR Biomed..

[5] Guilarte T.R. (2010). Manganese and Parkinson’s Disease: A Critical Review and New Findings. Environ. Health Perspect..

[6] Sanders T., Liu Y., Buchner V., Tchounwou P.B. (2009). Neurotoxic Effects and Biomarkers of Lead Exposure: A Review. Rev. Environ. Health.

[7] Wani A.L., Ara A., Usmani J.A. (2015). Lead Toxicity: A Review. Interdiscip. Toxicol..

[8] McKnight R.F., Adida M., Budge K., Stockton S., Goodwin G.M., Geddes J.R. (2012). Lithium Toxicity Profile: A Systematic Review and Meta-Analysis. Lancet.

[9] Godt J., Scheidig F., Grosse-Siestrup C., Esche V., Brandenburg P., Reich A., Groneberg D.A. (2006). The Toxicity of Cadmium and Resulting Hazards for Human Health. J. Occup. Med. Toxicol..

[10] Genchi G., Sinicropi M.S., Lauria G., Carocci A., Catalano A. (2020). The Effects of Cadmium Toxicity. Int. J. Environ. Res. Public Health.

[11] Leyssens L., Vinck B., Van Der Straeten C., Wuyts F., Maes L. (2017). Cobalt Toxicity in Humans—A Review of the Potential Sources and Systemic Health Effects. Toxicology.

[12] Simonsen L.O., Harbak H., Bennekou P. (2012). Cobalt Metabolism and Toxicology—A Brief Update. Sci. Total Environ..

[13] Buxton S., Garman E., Heim K.E., Lyons-Darden T., Schlekat C.E., Taylor M.D., Oller A.R. (2019). Concise Review of Nickel Human Health Toxicology and Ecotoxicology. Inorganics.

[14] Genchi G., Carocci A., Lauria G., Sinicropi M.S., Catalano A. (2020). Nickel: Human Health and Environmental Toxicology. Int. J. Environ. Res. Public Health.

[15] Flora G., Gupta D., Tiwari A. (2012). Toxicity of Lead: A Review with Recent Updates. Interdiscip. Toxicol..

[16] Lei T., Jing Y., Lv W., Li Y., Wang N., Wang J. (2026). Recovering Critical Elements from Wastewater for Battery Materials: A Review. Rare Met..

[17] Zha F., Wang S., Liu Z., Dai J., Yue S., Qi W., Xue X., Wang X., Zhang S. (2024). Removal of Heavy Metals from Fly Ash Using Electrodialysis Driven by a Bioelectrochemical System: A Case Study of Pb, Mn, Cu and Cd. Environ. Technol..

[18] Kapepula V.L., Luis P. (2024). Removal of Heavy Metals from Wastewater Using Reverse Osmosis. Front. Chem. Eng..

[19] Castro K., Abejón R. (2024). Removal of Heavy Metals from Wastewaters and Other Aqueous Streams by Pressure-Driven Membrane Technologies: An Outlook on Reverse Osmosis, Nanofiltration, Ultrafiltration and Microfiltration Potential from a Bibliometric Analysis. Membranes.

[20] Koli M.M., Singh S.P. (2023). Surface-Modified Ultrafiltration and Nanofiltration Membranes for the Selective Removal of Heavy Metals and Inorganic Groundwater Contaminants: A Review. Environ. Sci. Water Res. Technol..

[21] Koopman C., Witkamp G.J. (1999). Removal of Heavy Metals by Solvent Extraction and Supported Solvent Extraction during a Recrystallization Step in the Phosphoric Acid Production Process. Proceedings of the Engineering Foundation Conference.

[22] Chen S., Pei X., Huang Y., Luo Y., Xu H., Feng L., Liu W., Liu X. (2026). From Removal Claims to Engineering Evidence: Electrochemical Treatment and Selective Recovery of Heavy Metals in Wastewater. Nanoscale.

[23] Benalia M.C., Youcef L., Bouaziz M.G., Achour S., Menasra H. (2022). Removal of Heavy Metals from Industrial Wastewater by Chemical Precipitation: Mechanisms and Sludge Characterization. Arab. J. Sci. Eng..

[24] Abdullah A.S., Al-Asadi A.A., Ayoob H.W., Mohammed H.H., Homod R.Z., Mohammad H.I. (2026). Critical Review of Adsorption Technologies for Heavy Metal Removal: Mechanistic, Computational, and Policy Perspectives. RSC Adv..

[25] Dotto G.L., McKay G. (2020). Current Scenario and Challenges in Adsorption for Water Treatment. J. Environ. Chem. Eng..

[26] Sherpa S.W., Ponnuchamy M., Jacob M.M., Kapoor A. (2026). Exploring Bio-Based Sorbents for Efficient Removal of Hazardous Ionic Pollutants: A Comprehensive Review. Chem. Pap..

[27] Vasić V., Kukić D., Šćiban M., Đurišić-Mladenović N., Velić N., Pajin B., Crespo J., Farre M., Šereš Z. (2023). Lignocellulose-Based Biosorbents for the Removal of Contaminants of Emerging Concern (CECs) from Water: A Review. Water.

[28] Saraswat P., Singh S., Prasad M., Misra R., Rajput V.D., Ranjan R. (2023). Applications of Bio-Based Nanomaterials in Environment and Agriculture: A Review on Recent Progresses. Hybrid. Adv..

[29] Khoo P.S., Ilyas R.A., Aiman A., Wei J.S., Yousef A., Anis N., Zuhri M.Y.M., Abral H., Sari N.H., Syafri E. (2024). Revolutionizing Wastewater Treatment: A Review on the Role of Advanced Functional Bio-Based Hydrogels. Int. J. Biol. Macromol..

[30] Pratama A.W., Widiastuti N., Asranudin, Holilah, Firmanda A., Zulfiani U., Hanifah A., Piluharto B., El Messaoudi N. (2026). Structure-Property Relationships of Functionalized Cellulose Nanofibers and Their Composites for Heavy Metal Ions and Dye Removal: A Review. Polymer.

[31] Baraka F., Ganesan K., Milow B., Labidi J. (2024). Cellulose Nanofiber Aerogels: Effect of the Composition and the Drying Method. Cellulose.

[32] Fang Q., Sun H.-N., Zhang M., Mu T.-H., Garcia-Vaquero M. (2025). Cellulose Nanofibers: Current Status and Emerging Development of Sources, Pretreatment, Production, and Applications. ACS Agric. Sci. Technol..

[33] Turner T., Wheeler R., Oliver I.W. (2022). Evaluating Land Application of Pulp and Paper Mill Sludge: A Review. J. Environ. Manag..

[34] Hassan M.L., Hassan E.A., Elseoud W.S.A., Moustafa A.M. (2025). Utilization of Paper Sludge in Preparation of High-Purity Calcium Formate. Biomass Convers. Biorefin..

[35] El-Mekkawi S., Abou-Elseoud W., Fadel S., Hassan E., Hassan M. (2024). Phosphoric Acid Pretreatment and Saccharification of Paper Sludge as a Renewable Material for Cellulosic Fibers. J. Renew. Mater..

[36] Hassan E., Abou-Elseoud W., El-Mekkawi S., Hassan M. (2025). Saccharification of Paper Sludge and Fiber Dust Wastes from the Tissue Paper Industry by Maximyze^®^ Enzymes. J. Renew. Mater..

[37] Kuchangi N.S., Divakar S.A., Ajjipura B.H., Akhil B., Billava R.V., Shivanand B., Meenakshi S.I. (2026). Biopolymer and Cellulose-Based Aerogels: The Next-Generation Porous Materials for Sustainable Applications. Sustain. Polym. Energy.

[38] Parit M., Du H., Zhang X., Jiang Z. (2022). Flexible, Transparent, UV-Protecting, Water-Resistant Nanocomposite Films Based on Polyvinyl Alcohol and Kraft Lignin-Grafted Cellulose Nanofibers. ACS Appl. Polym. Mater..

[39] Sarder R., Starrett N., Agate S., Pal L. (2024). Decarbonizing Paper Mill Sludge Waste into Micro and Nanofibrillated Cellulose via Enzyme Hydrolysis and Dual Asymmetric Centrifugation. Waste Manag..

[40] Mautner A., Maples H.A., Kobkeatthawin T., Kokol V., Karim Z., Li K., Bismarck A. (2016). Phosphorylated Nanocellulose Papers for Copper Adsorption from Aqueous Solutions. Int. J. Environ. Sci. Technol..

[41] Li J., Alamdari N.E., Aksoy B., Parit M., Jiang Z. (2023). Integrated Enzyme Hydrolysis Assisted Cellulose Nanofibril (CNF) Fabrication: A Sustainable Approach to Paper Mill Sludge (PMS) Management. Chemosphere.

[42] Gallo Stampino P., Riva L., Caruso M., Rahman I.A., Elegir G., Bussini D., Marti-Rujas J., Dotelli G., Punta C. (2022). Can TEMPO-Oxidized Cellulose Nanofibers Be Used as Additives in Bio-Based Building Materials? A Preliminary Study on Earth Plasters. Materials.

[43] Adu C., Berglund L., Oksman K., Eichhorn S.J., Jolly M., Zhu C. (2018). Properties of Cellulose Nanofibre Networks Prepared from Never-Dried and Dried Paper Mill Sludge. J. Clean. Prod..

[44] Adu C., Rahatekar S., Filby J., Ayre D., Jolly M. (2019). Structural Packaging Foams Prepared by Uni-Directional Freezing of Paper Sludge Cellulose Nanofibres and Poly (Vinyl Alcohol). Mater. Lett..

[45] Du H., Parit M., Wu M., Che X., Wang Y., Zhang M., Wang R., Zhang X., Jiang Z., Li B. (2020). Sustainable Valorization of Paper Mill Sludge into Cellulose Nanofibrils and Cellulose Nanopaper. J. Hazard. Mater..

[46] Altay B.N., Huq A., Aksoy B., Hailstone R., Demir M., Aydemir C., Karademir A., Williams S. (2023). Enhanced Internal Coating Structure and Light Reflectance of Coated Papers: A Sludge Valorization Process. ACS Sustain. Chem. Eng..

[47] Saito T., Kimura S., Nishiyama Y., Isogai A. (2007). Cellulose Nanofibers Prepared by TEMPO-Mediated Oxidation of Native Cellulose. Biomacromolecules.

[48] Isogai A., Hänninen T., Fujisawa S., Saito T. (2018). Review: Catalytic Oxidation of Cellulose with Nitroxyl Radicals under Aqueous Conditions. Prog. Polym. Sci..

[49] French A.D. (2014). Idealized Powder Diffraction Patterns for Cellulose Polymorphs. Cellulose.

[50] Dewi R., Agusnar H., Alfian Z., Tamrin (2018). Characterization of Technical Kaolin Using XRF, SEM, XRD, FTIR and Its Potentials as Industrial Raw Materials. J. Phys. Conf. Ser..

[51] Liu B., Sun H., Peng T., He Q. (2018). One-Step Synthesis of Hydroxysodalite Using Natural Bentonite at Moderate Temperatures. Minerals.

[52] Mahfoudhi N., Boufi S. (2017). Nanocellulose as a Novel Nanostructured Adsorbent for Environmental Remediation: A Review. Cellulose.

[53] Qasem N.A.A., Mohammed R.H., Lawal D.U. (2021). Removal of Heavy Metal Ions from Wastewater: A Comprehensive and Critical Review. npj Clean Water.

[54] Kunz Lazzari L., Perondi D., Zattera A.J., Campomanes Santana R.M. (2021). Cellulose/Biochar Cryogels: A Study of Adsorption Kinetics and Isotherms. Langmuir.

[55] Saah F., Nagpal G., Gbawoquiyq F., Chaudhary R. (2024). Groundnut Shell Carbon Quantum Dot Magnetic Iron Oxide Nanocomposite (GSCQD-FeFe_2_O_4_) for Lead Removal from Water. Zast. Mater..

[56] Zhao D., Deng H., Wang W., Hu L., Ye S., Fu J., Zhang S. (2025). Synthesis, Characterization and Adsorption of Pb(II), Cd(II) and Cu(II) by Red Mud/Polyacrylic Acid/Sodium Carboxymethyl Cellulose Hydrogel. Arab. J. Chem..

[57] Khafagy E.-S., Alshahrani N., Lila A.S.A., Al Saqr A., Aldawsari M.F., Almutairy B.K., Mostafa M.A., Ashmawy A.M. (2026). Design, Synthesis, and Characterization of Glyoxime-Functionalized Hydroxypropyl Methylcellulose/Acrylic Acid Hydrogel for Efficient Removal of Lead (II) Ions from Aqueous Media. Sci. Rep..

[58] Li Z., Qiu J., Xu X., Wan R., Yao M., Wang H., Zhou Z., Xu J. (2025). Solar Driven Kaolin-Based Hydrogels for Efficient Interfacial Evaporation and Heavy Metal Ion Adsorption from Wastewater. Sep. Purif. Technol..

[59] Chen J., Wang X., Huang X., Tong Z., Zhou J., Shen Y., Hao C. (2025). Construction of Cellulose-Based Hydrogel Compounded with Modified Kaolin and Its Removal Performance for Heavy Metal Ions and Dyes in Water. Int. J. Biol. Macromol..

[60] Yang M., Lei N., Liu J., Yang L., Li B., He X. (2018). Removal of Heavy Metal Ions from Wastewater by Combined Modified Kaolin. Desalin. Water Treat..

[61] Sone A., Saito T., Isogai A. (2016). Preparation of Aqueous Dispersions of TEMPO-Oxidized Cellulose Nanofibrils with Various Metal Counterions and Their Super Deodorant Performances. ACS Macro Lett..

[62] Paul H.R., Bera M.K., Macke N., Rowan S.J., Tirrell M.V. (2024). Quantitative Determination of Metal Ion Adsorption on Cellulose Nanocrystals Surfaces. ACS Nano.

[63] Ambikadevi V.R., Lalithambika M. (2000). Effect of Organic Acids on Ferric Iron Removal from Iron-Stained Kaolinite. Appl. Clay Sci..

[64] Chai J.-B., Au P.-I., Mubarak N.M., Khalid M., Ng W.P.-Q., Jagadish P., Walvekar R., Abdullah E.C. (2020). Adsorption of Heavy Metal from Industrial Wastewater onto Low-Cost Malaysian Kaolin Clay–Based Adsorbent. Environ. Sci. Pollut. Res..

[65] Narwade V.N., Khairnar R.S., Kokol V. (2018). In Situ Synthesized Hydroxyapatite—Cellulose Nanofibrils as Biosorbents for Heavy Metal Ions Removal. J. Polym. Environ..

[66] Xu C., Yu T., Peng J., Zhao L., Li J., Zhai M. (2020). Efficient Adsorption Performance of Lithium Ion onto Cellulose Microspheres with Sulfonic Acid Groups. Quantum Beam Sci..

[67] Zheng X., Wang Y., Qiu F., Li Z., Yan Y. (2019). Dual-Functional Mesoporous Films Templated by Cellulose Nanocrystals for the Selective Adsorption of Lithium and Rubidium. J. Chem. Eng. Data.

[68] Al-Ghouti M.A., Da’ana D.A. (2020). Guidelines for the Use and Interpretation of Adsorption Isotherm Models: A Review. J. Hazard. Mater..

[69] Wang J., Guo X. (2020). Adsorption Isotherm Models: Classification, Physical Meaning, Application and Solving Method. Chemosphere.

[70] Hubbe M.A., Beck K.R., O’Neal W.G., Sharma Y.C. (2012). Cellulosic substrates for removal of pollutants from aqueous systems: A review. 2. Dyes. Bioresources.

[71] Amirjani A., Kamani P., Hosseini H.R.M., Sadrnezhaad S.K. (2022). SPR-Based Assay Kit for Rapid Determination of Pb^2+^. Anal. Chim. Acta.

[72] Sangoremi A.A. (2025). Adsorption Kinetic Models and Their Applications: A Critical Review. Int. J. Res. Sci. Innov..

[73] Hubbe M.A., Wu N., Rojas O.J., Park S. (2019). Implications of Apparent Pseudo-Second-Order Adsorption Kinetics onto Cellulosic Materials: A Review. Bioresources.

[74] Tran H.N., You S.-J., Hosseini-Bandegharaei A., Chao H.-P. (2017). Mistakes and Inconsistencies Regarding Adsorption of Contaminants from Aqueous Solutions: A Critical Review. Water Res..

[75] Anastopoulos I., Kyzas G.Z. (2016). Are the Thermodynamic Parameters Correctly Estimated in Liquid-Phase Adsorption Phenomena?. J. Mol. Liq..

[76] Syeda H.I., Sultan I., Razavi K.S., Yap P.-S. (2022). Biosorption of Heavy Metals from Aqueous Solution by Various Chemically Modified Agricultural Wastes: A Review. J. Water Process Eng..

[77] Zhong Y., An Y., Wang K., Zhang W., Hu Z., Chen Z., Wang S., Wang B., Wang X., Li X. (2022). Evaluation of Aerogel Spheres Derived from Salix Psammophila in Removal of Heavy Metal Ions in Aqueous Solution. Forests.

[78] Hernández-Guerrero B.A., Martínez L., Peña-Rodríguez G., Trejo F. (2026). Machine Learning-Enhanced Modeling of Heavy Metal Adsorption onto Coal Fly Ash-Derived Zeolite P. Water.

[79] Kim H., Mattinen U., Guccini V., Liu H., Salazar-Alvarez G., Lindström R.W., Lindbergh G., Cornell A. (2020). Feasibility of Chemically Modified Cellulose Nanofiber Membranes as Lithium-Ion Battery Separators. ACS Appl. Mater. Interfaces.

[80] Gosselin A.J., Rowland C.A., Balto K.P., Yap G.P.A., Bloch E.D. (2018). Design and Synthesis of Porous Nickel(II) and Cobalt(II) Cages. Inorg. Chem..

[81] Godwin J., Njimou J.R., Abdus-Salam N., Adegoke H.I., Panda P.K., Tripathy B.C., Maicaneanu S.A. (2023). Nanosorbent Based on Coprecipitation of ZnO in Goethite for Competitive Sorption of Cd(II)-Pb(II) and Cd(II)-Pb(II)-Ni(II) Systems. J. Environ. Health Sci. Eng..

[82] Lazar M.M., Ghiorghita C.-A., Dragan E.S., Humelnicu D., Dinu M.V. (2023). Ion-Imprinted Polymeric Materials for Selective Adsorption of Heavy Metal Ions from Aqueous Solution. Molecules.

[83] Wang Z., Li W., Wang L., Zhang Y., Li J., Liu Y. (2023). Adsorption of Zn(II), Pb(II), and Cu(II) by Residual Soil-Derived Zeolite in Single-Component and Competitive Systems. Sustainability.

[84] Mahpouya F., Burris C.J., Paul H., Nikolaev A. (2026). Maximizing the Expected Value of Experimentation for Finding Top-κ Rank *via* Aggregation of Pairwise Comparisons. IISE Trans..

[85] Saito T., Isogai A. (2004). TEMPO-Mediated Oxidation of Native Cellulose. The Effect of Oxidation Conditions on Chemical and Crystal Structures of the Water-Insoluble Fractions. Biomacromolecules.

[86] Segal L., Creely J.J., Martin A.E., Conrad C.M. (1959). An Empirical Method for Estimating the Degree of Crystallinity of Native Cellulose Using the X-Ray Diffractometer. Text. Res. J..

[87] Vogel A.I., Jeffery G.H. (1989). Vogel’s Textbook of Quantitative Chemical Analysis.

