# Supplementary material for: Preparation of Cellulose Nanofiber Aerogels from Paper Sludge Waste and Their Use for the Removal of Metal Ions Used in Batteries from Water
Source: Gels. 2026 Oct 17;12(10):935. doi: 10.3390/gels12100935 (PMC13704091; doi:10.3390/gels12100935)
Supplement: Supplementary file 1 [file gels-12-00935-s001.zip › gels-4579637-supplementary.pdf]

Quantachrome TouchWin™

version 1.21

Copyright ©1994-2015 Quantachrome Instruments

**Report date:**	Mon Jun 22 2026	**Operator:**	nrc
**Filename:**	CB 16062026.qcuPhysIso		

Analysis Information

Sample

**ID**	CB 16062026	**Weight**	0.0174 g		
**Description**					

Analysis

**Data ID**	{909f431a-b4b4-430b-9967-43e42ff13ab0}				
**Operator**	nrc	**Date**	2001.12.31	**Duration**	256.967 min
**Instrument**	St 2 on NOVA touch 4LX [s/n:17016062702]			**Firmware**	1.04
**Comments**					
**Ambient Temp.**	31.0337 °C	**Void Volume Mode**	He Measure	**Cold Zone**	4.60306 cc
**Warm Zone**	7.73776 cc	**Cell Type**	9mm with rod	**Thermal Delay**	120 sec
**Po Mode**	Calculate				

Adsorbate

**Name**	Nitrogen	**Molecular Weight**	28.013 g/mol	**Cross Section Area**	16.2 Å²/mol
**Non-ideality**	6.58e-005 1/Torr	**Bath Temperature**	77.35 K		

Degas information

**Time**	0 hours	**Temp**	0 °C

Data Reduction Parameters

Thermal Transpiration no

Adsorbate Model

**Name**	Nitrogen	**Molecular Weight**	28.0134 g	**Cross Section Area**	16.2 Å²/molec
**Bath Temperature**	77.35 K				

BET Multi-point BET results

**Isotherm Branch**	Adsorption
**Slope**	-1.40839
**Intercept**	14.2559
**Correlation coeff., r**	0.530987
**C constant**	0.901207
**Surface area**	271.065 m²/g

Graph - BET Multi-point BET

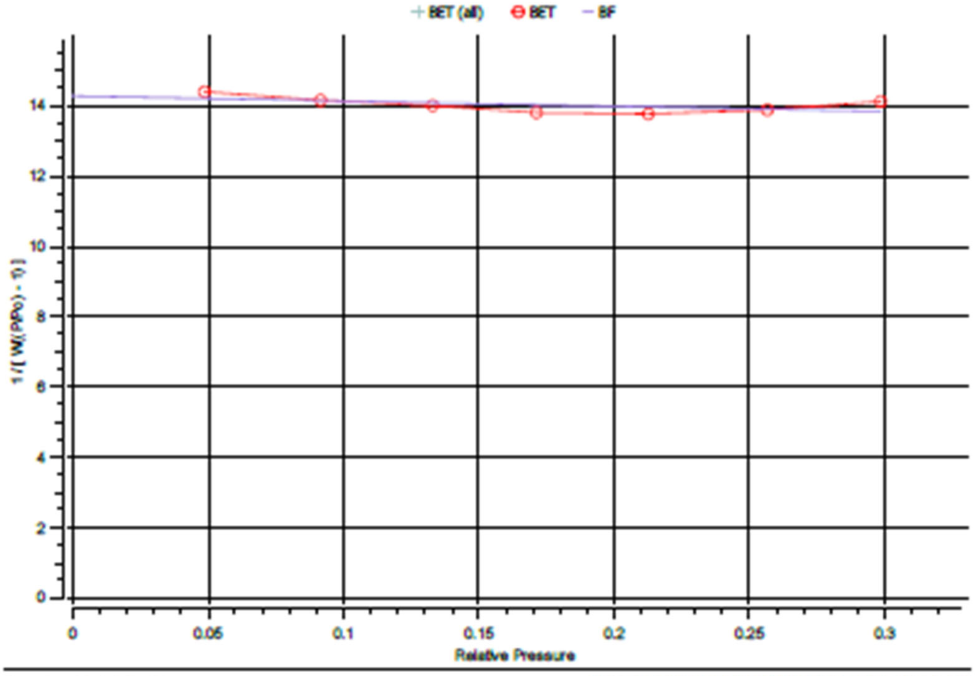

Quantachrome TouchWin™

version 1.21

Copyright ©1994-2015 Quantachrome Instruments

Report date: Mon Jun 22 2026 **Operator:** nrc
Filename: CB 16062026.qcuPhysIso

Analysis Information

Sample
ID CB 16062026 **Weight** 0.0174 g
Description
Analysis
Data ID {909f431a-b4b4-430b-9967-43e42ff13ab0}
Operator nrc **Date** 2001.12.31 **Duration** 256.967 min
Instrument St 2 on NOVA touch 4LX [s/n:17016062702] **Firmware** 1.04
Comments
Ambient Temp. 31.0337 °C **Void Volume Mode** He Measure **Cold Zone** 4.60306 mL
Warm Zone 7.73776 mL **Cell Type** 9mm with rod **Thermal Delay** 120 sec
Po Mode Calculate
Adsorbate
Name Nitrogen **Molecular Weight** 28.013 g/mol **Cross Section Area** 16.2 Å²/mol
Non-ideality 6.58e-005 1/torr **Bath Temperature** 77.35 K
Degas information
Time 0 hours **Temp** 0 °C

Data Reduction Parameters

Thermal Transpiration no
Temp. Comp no
Thickness Method deBoer
Adsorbate Model
Name Nitrogen **Molecular Weight** 28.0134 g **Cross Section Area** 16.2 Å²/molec
Bath Temperature 77.35 K

Total Pore Volume results

Total Pore Volume 1.5140e-001 cc/g
for pores smaller than 244.14 nm (radius)
at relative pressure 0.99606

Quantachrome TouchWin™

version 1.21

Copyright ©1994-2015 Quantachrome Instruments

Report date: Mon Jun 22 2026 **Operator:** nrc
Filename: CB 16062026.qcuPhysIso

Analysis Information

Sample
ID CB 16062026 **Weight** 0.0174 g
Description
Analysis
Data ID {909f431a-b4b4-430b-9967-43e42ff13ab0}
Operator nrc **Date** 2001.12.31 **Duration** 256.967 min
Instrument St 2 on NOVA touch 4LX [s/n:17016062702] **Firmware** 1.04
Comments
Ambient Temp. 31.0337 °C **Void Volume Mode** He Measure **Cold Zone** 4.60306 mL
Warm Zone 7.73776 mL **Cell Type** 9mm with rod **Thermal Delay** 120 sec
Po Mode Calculate
Adsorbate
Name Nitrogen **Molecular Weight** 28.013 g/mol **Cross Section Area** 16.2 Å²/mol
Non-ideality 6.58e-005 1/torr **Bath Temperature** 77.35 K
Degas information
Time 0 hours **Temp** 0 °C

Data Reduction Parameters

Thermal Transpiration no
Temp. Comp no
Thickness Method deBoer
Adsorbate Model
Name Nitrogen **Molecular Weight** 28.0134 g **Cross Section Area** 16.2 Å²/molec
Bath Temperature 77.35 K

Average Pore Size results

Average Pore radius 1.1171e+000 nm

Supplementary Figure S1. Surface area BET curves, total pore volume, and average pore radium of PS-CNF aerogel.

Quantachrome TouchWin™

version 1.21

Copyright ©1994-2015 Quantachrome Instruments

**Report date:**	Mon Jun 22 2026	**Operator:**	nrc
**Filename:**	CW 16062026.qcuPhysIso		

Analysis Information

Sample

**ID**	CW 16062026	**Weight**	0.0188 g		
**Description**					

Analysis

**Data ID**	{595a3917-186e-490c-9cbd-64056afe34a7}				
**Operator**	nrc	**Date**	2001.12.31	**Duration**	268.383 min
**Instrument**	St 1 on NOVA touch 4LX [s/n:17016062702]			**Firmware**	1.04
**Comments**					
**Ambient Temp.**	30.9755 °C	**Void Volume Mode**	He Measure	**Cold Zone**	4.64496 mL
**Warm Zone**	7.82108 mL	**Cell Type**	9mm with rod	**Thermal Delay**	120 sec
**Po Mode**	Calculate				

Adsorbate

**Name**	Nitrogen	**Molecular Weight**	28.013 g/mol	**Cross Section Area**	16.2 Å²/mol
**Non-ideality**	6.58e-005 1/torr	**Bath Temperature**	77.35 K		

Degas information

**Time**	0 hours	**Temp**	0 °C

Data Reduction Parameters

**Thermal Transpiration**	no

Adsorbate Model

**Name**	Nitrogen	**Molecular Weight**	28.0134 g	**Cross Section Area**	16.2 Å²/molec
**Bath Temperature**	77.35 K				

BET Multi-point BET results

**Isotherm Branch**	Adsorption
**Slope**	-1.80985
**Intercept**	14.7384
**Correlation coeff., r**	0.452369
**C constant**	0.877202
**Surface area**	269.366 m²/g

Graph - BET Multi-point BET

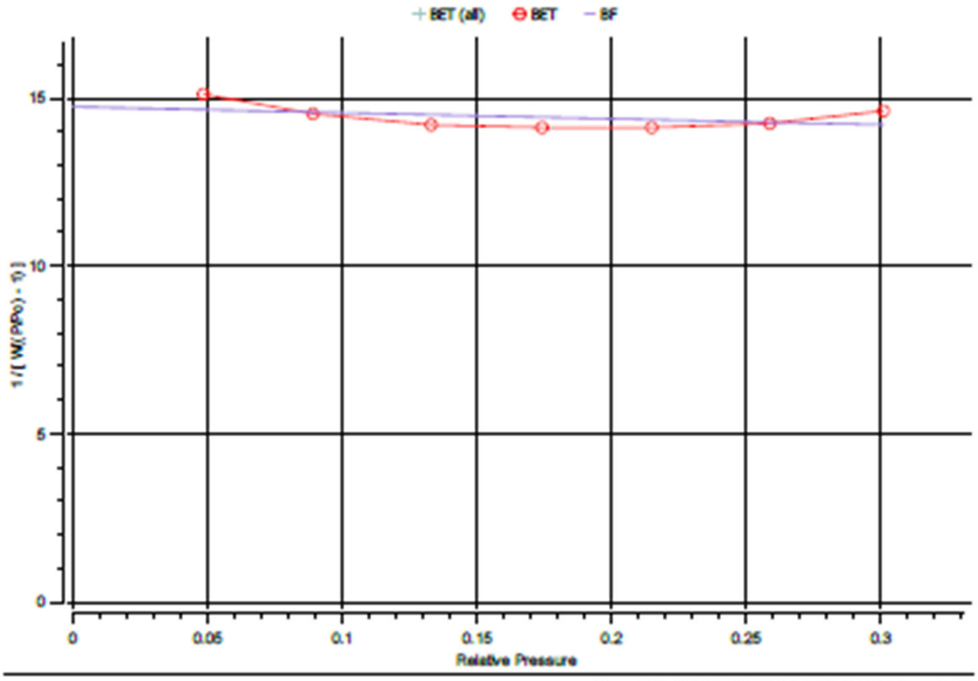

Quantachrome TouchWin™

version 1.21

Copyright ©1994-2015 Quantachrome Instruments

Report date: Mon Jun 22 2026 **Operator:** nrc
Filename: CW 16062026.qcuPhysIso

Analysis Information

Sample
ID CW 16062026 **Weight** 0.0188 g
Description
Analysis
Data ID {595a3917-186e-490c-9cbd-64056afe34a7}
Operator nrc **Date** 2001.12.31 **Duration** 268.383 min
Instrument St 1 on NOVA touch 4LX [s/n:17016062702] **Firmware** 1.04
Comments
Ambient Temp. 30.9755 °C **Void Volume Mode** He Measure **Cold Zone** 4.64496 mL
Warm Zone 7.82108 mL **Cell Type** 9mm with rod **Thermal Delay** 120 sec
Po Mode Calculate
Adsorbate
Name Nitrogen **Molecular Weight** 28.013 g/mol **Cross Section Area** 16.2 Å²/mol
Non-ideality 6.58e-005 1/torr **Bath Temperature** 77.35 K
Degas information
Time 0 hours **Temp** 0 °C

Data Reduction Parameters

Thermal Transpiration no
Temp. Comp no
Thickness Method deBoer
Adsorbate Model
Name Nitrogen **Molecular Weight** 28.0134 g **Cross Section Area** 16.2 Å²/molec
Bath Temperature 77.35 K

Total Pore Volume results

Total Pore Volume 1.4043e-001 cc/g
for pores smaller than 352.62 nm (radius)
at relative pressure 0.99728

Quantachrome TouchWin™

version 1.21

Copyright ©1994-2015 Quantachrome Instruments

Report date: Mon Jun 22 2026 **Operator:** nrc
Filename: CW 16062026.qcuPhysIso

Analysis Information

Sample
ID CW 16062026 **Weight** 0.0188 g
Description
Analysis
Data ID {595a3917-186e-490c-9cbd-64056afe34a7}
Operator nrc **Date** 2001.12.31 **Duration** 268.383 min
Instrument St 1 on NOVA touch 4LX [s/n:17016062702] **Firmware** 1.04
Comments
Ambient Temp. 30.9755 °C **Void Volume Mode** He Measure **Cold Zone** 4.64496 mL
Warm Zone 7.82108 mL **Cell Type** 9mm with rod **Thermal Delay** 120 sec
Po Mode Calculate
Adsorbate
Name Nitrogen **Molecular Weight** 28.013 g/mol **Cross Section Area** 16.2 Å²/mol
Non-ideality 6.58e-005 1/torr **Bath Temperature** 77.35 K
Degas information
Time 0 hours **Temp** 0 °C

Data Reduction Parameters

Thermal Transpiration no
Temp. Comp no
Thickness Method deBoer
Adsorbate Model
Name Nitrogen **Molecular Weight** 28.0134 g **Cross Section Area** 16.2 Å²/molec
Bath Temperature 77.35 K

Average Pore Size results

Average Pore radius 1.0427e+000 nm

Supplementary Figure S2. Surface area BET curves, total pore volume, and average pore radium of FD-CNF aerogel.
